# Artificial Intelligence–Enabled Imaging for Predicting Preoperative Extraprostatic Extension in Prostate Cancer: Systematic Review and Meta-Analysis

**DOI:** 10.2196/80981

**Published:** 2025-12-09

**Authors:** Xin Zhang, Yiting Qi, Xi Wang, Haowen Chen, Jia Li

**Affiliations:** 1 Department of Nursing, Zhuhai Campus of Zunyi Medical University, 368 Jinhaian Community, Sanzao Town, Jinwan District, Zhuhai, Guangdong Province, 519000, China, 86 137 2625 6630; 2 Department of Ultrasound Imaging, Zhuhai People's Hospital, The Affiliated Hospital of Beijing Institute of Technology, Zhuhai Clinical Medical College of Jinan University, Zhuhai, Guangdong, China; 3 Department of Nursing, Kiang Wu Nursing College of Macau, Macau, China

**Keywords:** artificial intelligence, extraprostatic extension, prostate cancer, magnetic resonance imaging, meta-analysis

## Abstract

**Background:**

Artificial intelligence (AI) techniques, particularly those using machine learning and deep learning to analyze multimodal imaging data, have shown considerable promise in enhancing preoperative prediction of extraprostatic extension (EPE) in prostate cancer.

**Objective:**

This meta-analysis compares the diagnostic performance of AI-enabled imaging techniques with that of radiologists for predicting preoperative EPE in prostate cancer.

**Methods:**

We conducted a systematic literature search in PubMed, Embase, and Web of Science up to September 2025, following PRISMA-DTA (Preferred Reporting Items for Systematic Reviews and Meta-Analysis of Diagnostic Test Accuracy) guidelines. Studies applying AI techniques to predict EPE using multiparametric magnetic resonance imaging (mpMRI) and prostate-specific membrane antigen positron emission tomography (PSMA PET) imaging were included. Sensitivity, specificity, and area under the curve (AUC) for both internal and external validation sets were extracted and pooled using a bivariate random effects model. Study quality was assessed using the modified Quality Assessment of Diagnostic Performance Studies (QUADAS-2) tool.

**Results:**

A total of 21 studies were included in the analysis. For internal validation sets in patient-based analyses, mpMRI-based AI demonstrated a pooled sensitivity of 0.77 (95% CI 0.71-0.82), specificity of 0.71 (95% CI 0.64-0.78), and AUC of 0.81 (95% CI 0.77-0.84). In external validation, mpMRI-based AI achieved a sensitivity of 0.66 (95% CI 0.43-0.84), specificity of 0.80 (95% CI 0.64-0.90), and AUC of 0.80 (95% CI 0.77-0.84). In comparison, radiologists achieved a pooled sensitivity of 0.69 (95% CI 0.60-0.76), specificity of 0.73 (95% CI 0.66-0.78), and AUC of 0.77 (95% CI 0.73-0.80). Statistical comparisons between mpMRI-based AI and radiologists showed no significant difference in sensitivity (*Z*=1.61; *P*=.10), specificity (*Z*=0.43; *P*=.67)*.* Conversely, the AUC of mpMRI-based AI was significantly higher than that of PSMA PET-based (*Z*=2.77; *P=*.01)*.* PSMA PET-based AI showed moderate performance with sensitivity of 0.73 (95% CI 0.65-0.80), specificity of 0.61 (95% CI 0.30-0.85), and AUC of 0.74 (95% CI 0.70-0.77) in internal validation, and in external validation, it demonstrated sensitivity of 0.77 (95% CI 0.57-0.89) and specificity of 0.50 (95% CI 0.22-0.78), demonstrating no significant advantage over radiologists.

**Conclusions:**

mpMRI-based AI demonstrated improved diagnostic performance for preoperative prediction of EPE in prostate cancer compared to conventional radiological assessment, achieving higher AUC. However, PSMA PET-based AI models currently offer no significant advantage over either mpMRI-based AI or radiologists. Limitations include the retrospective design and high heterogeneity, which may introduce bias and affect generalizability. Larger, more diverse cohorts are essential for confirming these findings and optimizing the integration of AI in clinical practice.

## Introduction

The global incidence of prostate cancer (PCa) varies significantly across different regions. According to 2020 global data, the age-standardized incidence rate of PCa is 31 per 100,000 people, with a lifetime cumulative risk of 3.9% [1,2]. Several clinicopathological indicators are commonly used to assess PCa aggressiveness and guide treatment, including prostate-specific antigen levels, Gleason score, and tumor stage [3,4]. Among these, extraprostatic extension (EPE)—defined as tumor invasion beyond the prostatic capsule—serves as a distinct pathological hallmark of local disease progression [5]. Unlike prostate-specific antigen or Gleason score, which reflect biological activity or histological grade, EPE provides direct anatomical evidence of tumor spread, with important implications for staging and prognosis [6]. The presence of EPE significantly increases the risk of positive surgical margins, biochemical recurrence, distant metastasis, and cancer-specific mortality. Therefore, early and accurate detection of EPE is critical, as it informs clinical management, notably surgical planning, directly impacting patient outcomes, including recurrence rates and survival [7].

Conventional diagnostic modalities used for assessing EPE primarily include multiparametric magnetic resonance imaging (mpMRI), prostate-specific membrane antigen positron emission tomography (PSMA PET), and histopathological examination of biopsy or prostatectomy specimens [8]. mpMRI, as a noninvasive method, offers detailed visualization of the tumor microenvironment, yet its diagnostic performance is impeded by substantial inter- and intraobserver variability [7,9]. Moreover, traditional radiological evaluation relies predominantly on morphological features and quantitative imaging parameters that may not fully capture complex tumor heterogeneity that is obscured beyond human visual perception [10]. PSMA PET provides valuable metabolic information but suffers from limited availability and lacks standardized protocols [11]. Histopathological analysis remains the gold standard but is invasive and confined to postoperative confirmation, limiting its utility for preoperative decision-making [12].

Artificial intelligence (AI) techniques, particularly those using machine learning and deep learning (DL) to analyze multimodal imaging data, have shown considerable promise in enhancing preoperative prediction of EPE [13]. AI models can extract high-dimensional features from mpMRI and PSMA PET, potentially identifying subtle imaging patterns beyond human perception, thereby improving diagnostic precision and reproducibility [14]. Recent meta-analyses report pooled sensitivities and specificities for mpMRI radiomics–based models around 0.77 and 0.75 with area under the curve (AUC) values up to 0.91, reflecting moderate to high performance [12]. Nonetheless, significant controversies prevail concerning heterogeneity in diagnostic performance across external validation cohorts, discrepancies in performance among AI models trained on different imaging modalities, and the comparative effectiveness of AI versus experienced radiologists [15,16].

Therefore, this meta-analysis aimed to systematically synthesize current evidence on the diagnostic performance of AI-enabled imaging techniques versus radiologists for preoperative prediction of EPE in PCa, thereby clarifying their clinical utility and guiding future research directions.

## Methods

### Overview

The meta-analysis was conducted in strict accordance with the PRISMA-DTA (Preferred Reporting Items for Systematic Reviews and Meta-Analyses of Diagnostic Test Accuracy; Checklist 1) guidelines [17].

### Ethical Considerations

This is a systematic review and meta-analysis, and ethics approval and consent to participate were not applicable.

### Search Strategy

A comprehensive search of PubMed, Embase, and Web of Science databases was performed to identify relevant publications up to September 2025. This search used the following key terms: “Artificial intelligence,” “Machine learning,” “Deep learning,” “Extraprostatic extension in prostate cancer,” and “Prostate cancer.” A search strategy combining free-text keywords and Medical Subject Headings (MeSH) was used, with no restrictions on language or publication year during the initial search. Further details are available in Table S1 in Multimedia Appendix 1. Additionally, reference lists of the included studies were manually reviewed to uncover further pertinent articles.

### Inclusion and Exclusion Criteria

Studies were included based on the Participants, Index Test, Target Conditions, Reference Standard, Outcomes, Settings (PITROS) framework. Detailed inclusion criteria are presented in Table 1.

**Table 1.** Summary of inclusion criteria using the Participants, Index Test, Target Conditions, Reference Standard, Outcomes, Settings (PITROS) framework.

Criteria	Details
Participants (P)	Patients diagnosed with PCa^a^
Index test (I)	The study used AI^b^ techniques based on imaging (mpMRI^c^ and PSMA PET^d^) to predict the presence of the EPE^e^.
Target conditions (T)	The positive group was defined as patients finally diagnosed with EPE of PCa, whereas the negative group comprised patients ultimately diagnosed as negative for EPE. Studies that investigated or defined the target condition primarily based on perineural invasion were excluded.
Reference standard (R)	The reference standard was pathological biopsy.
Outcomes (O)	The primary outcomes included internal and external validation sets, radiologists’ sensitivity, specificity, and the area under the curve.
Settings (S)	The study setting was local hospitals with retrospective or prospective data sources.

a PCa: prostate cancer.

b AI: artificial intelligence.

c mpMRI: multiparametric magnetic resonance imaging.

d PSMA PET: prostate-specific membrane antigen positron emission tomography.

e EPE: extraprostatic extension.

Additionally, we excluded studies with irrelevant titles and abstracts, as well as certain document types, including reviews, case reports, conference abstracts, meta-analyses, and letters to the editor. Non-English studies and those not focused on EPE were excluded due to accessibility issues. Two reviewers (XZ and XW) independently screened titles and abstracts for relevance and then evaluated full texts based on the inclusion criteria. Duplicates were identified and removed using EndNote X9 software (Clarivate) duplicate detection tool, followed by manual verification. Discrepancies were resolved through discussion, and if no consensus was reached, a third reviewer (HC) made the final decision.

### Quality Assessment

We evaluated the quality of the included studies using the Quality Assessment of Diagnostic Performance Studies (QUADAS-2) tool [18], replacing some of the unrelated criteria with more relevant standards from another applicable tool (the Prediction Model Risk of Bias Assessment Tool) [19]. In this section, we describe the modified QUADAS-2 tool based on the experience of using the original tool and potential sources of bias arising from differences in the design and implementation of the included studies. Complete assessment details are provided in Table S2 in Multimedia Appendix 1.

The QUADAS-2 tool encompasses 4 essential domains: patient selection, index test (AI models), reference standard, and analysis. In addition to assessing the risk of bias in each of the 4 domains, we also evaluated concerns regarding applicability in 3 domains. Two reviewers (JL and XW) independently assessed the risk of bias in the included studies using a modified QUADAS-2 tool. Any discrepancies between the reviewers were resolved through discussion.

### Certainty of Evidence

The Grading of Recommendations, Assessment, Development, and Evaluations (GRADE) framework was used to assess the certainty of sensitivity and specificity in both internal and external validation sets [20], focusing on 5 key domains: risk of bias, indirectness, inconsistency, imprecision, and publication bias [20]. The certainty of evidence was downgraded whenever there was sufficient reason related to any of these domains. Full assessment details are provided in Table S3 in Multimedia Appendix 1.

### Data Extraction

Two reviewers (XZ and XW) independently extracted data from the full texts to determine study eligibility. Any disagreements were resolved through discussion with a third reviewer (HC) acting as an arbitrator. Extracted data included the first author’s name, publication year, study design, technical details (such as mpMRI sequences, reference standards, data splitting method, AI methods, and optimal AI algorithms), and study- and patient-level information (including training, internal, and external validation cohort sizes; patients’ country of origin; reference standards; analysis type; and the numbers of true positives [TP], true negatives [TN], false positives [FP], and false negatives [FN] in each dataset).

TP referred to cases classified as positive by the AI model based on imaging data and confirmed as EPE of PCa by pathological reference standards. TN denoted cases identified as negative by the AI model and confirmed by pathology as free of extracapsular PCa invasion. FP indicated cases classified as positive by the AI model but confirmed negative by reference standards (ie, misclassified benign lesions). FN referred to cases identified as negative by the AI model but confirmed positive by reference standards (ie, missed extracapsular PCa invasion). For studies included in the systematic review but lacking data suitable for meta-analysis, we contacted corresponding authors via email to obtain the necessary information.

As most studies did not provide diagnostic contingency tables, we used 3 main approaches to reconstruct the 2×2 tables: first, by calculating TP, FP, FN, and TN using reported sensitivity, specificity, number of positive cases per the reference standard, and total sample size; second, by digitizing receiver operating characteristic curves (ROCs) using GetData Graph Digitizer software (Informer Technologies, Inc), extracting optimal sensitivity and specificity based on the Youden index; and third, by estimating the TP, FP, FN, and TN counts using the reference standard positivity and total patient numbers [21].

### Outcome Measures

The primary outcome measures included sensitivity, specificity, and AUC in internal and external validation sets and among radiologists. Sensitivity (also known as recall or true positive rate) measures the ability of the AI model to accurately identify true cases, calculated as (TP)/(TP + FN). Specificity (also known as true negative rate) reflects the model’s ability to correctly identify healthy cases, calculated as TN/(TN + FP). AUC refers to the area under the ROC curve and is a comprehensive indicator of the model’s ability to distinguish between positive and negative cases. In studies providing multiple nonoverlapping patient data sources, we assumed these contingency tables to be independent and thus extracted them all. However, for studies presenting multiple AI models or models trained on different datasets, we only included the model with the best performance (ie, the highest AUC value) from the internal and external validation sets.

### Statistical Analysis

The bivariate random-effects meta-analysis was performed using the Reitsma model to synthesize the diagnostic performance metrics of AI systems and radiologists for detecting EPE of PCa [22]. We synthesized pooled sensitivity, specificity, and AUC values separately for mpMRI-based AI and PSMA-based AI across internal validation sets, external validation sets, and radiologists’ diagnostic results. For outcomes reported in 4 or more studies, summary ROC curves were generated to display the 95% CIs and prediction regions, where the prediction region illustrates the expected range of sensitivity and specificity in future studies [23].

To assess between-study heterogeneity, Higgins *I*^2^ statistic was calculated, with *I*^2^ values of 25%, 50%, and 75% representing low, moderate, and high heterogeneity, respectively [24]. Given that only the mpMRI-based internal validation set included more than 10 studies, subgroup analyses and meta-regression were conducted for this subset to explore potential sources of significant heterogeneity [25]. The meta-regression variables included imaging modality, geographic region, data partitioning strategy, regions of interest (ROI), analysis approach, and AI method. The comparative diagnostic performance between AI and radiologists was evaluated by obtaining independent pooled estimates for each group, followed by *z* tests of summary sensitivity, specificity, and AUC values. No continuity correction was applied, as no studies contained zero cells in diagnostic 2×2 tables. The clinical utility of the AI models and radiologists was further evaluated using Fagan nomograms. Publication bias was assessed via Deeks funnel plot asymmetry test, which uses a log-linear regression of the diagnostic odds ratio against effective sample size [26]. Statistical analyses were conducted using Stata version 15.1 with the Midas package, whereas risk of bias assessments were performed using the Cochrane Collaboration’s RevMan 5.4 software. A *P* value <.05 was considered statistically significant for all tests.

## Results

### Study Selection

**Figure 1.**
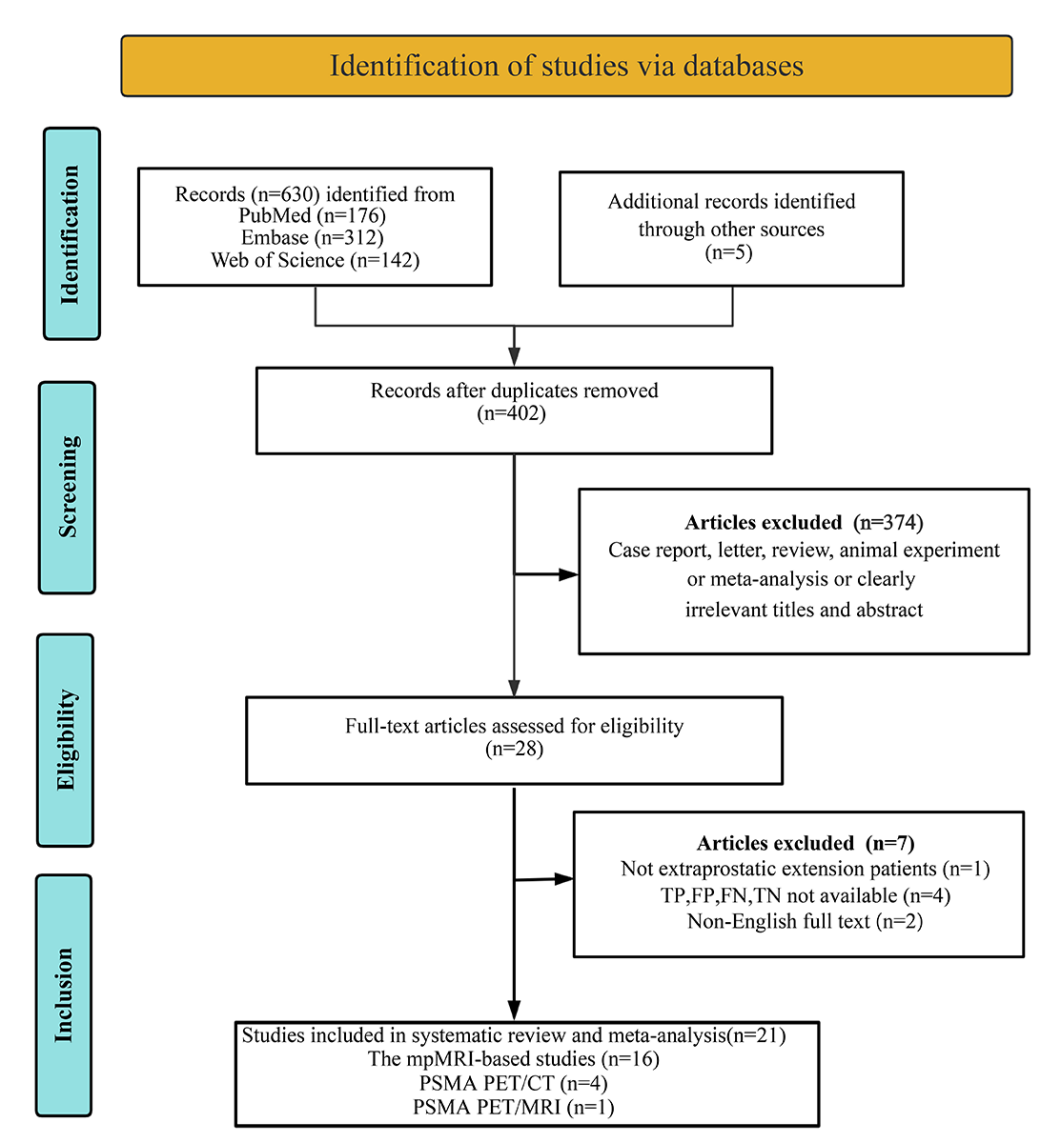
PRISMA (Preferred Reporting Items for Systematic Reviews and Meta-Analyses) flow diagram illustrating the study selection process. CT: computed tomography; FN: false negative; FP: false positive; mpMRI: multiparametric magnetic resonance imaging; MRI: magnetic resonance imaging; PSMA PET: prostate-specific membrane antigen positron emission tomography; TN: true negative; TP: true positive.

The initial database search yielded 630 potentially relevant articles, along with 5 (0.8%) additional articles from nondatabase sources. After removing 233 (37%) duplicate references, 402 (63%) articles remained for preliminary screening. During the initial screening phase, 374 (58%) articles were excluded because of clearly irrelevant titles and abstracts, as well as unsuitable publication types, resulting in 28 (4%) articles proceeding to full-text review. Following a thorough examination, 7 (1%) studies were excluded because of insufficient or incomplete diagnostic data (including TP, FP, FN, and TN), whereas 2 (0.3%) studies could not be acquired with full English text, and 1 (0.1%) study failed to evaluate EPE patients. Ultimately, 21 (3%) studies met the inclusion criteria and were incorporated into the meta-analysis [7,8,27-46]. The literature selection process adhered to the standardized PRISMA (Preferred Reporting Items for Systematic Reviews and Meta-Analyses) guidelines, as detailed in Figure 1.

### Study Description

A total of 21 eligible studies were identified, including 16 based on mpMRI [7,27-32,35,36,38,39,41,42,44-46], 4 based on PSMA PET/computed tomography (CT) [8,34,40,43], and 1 based on PSMA PET/magnetic resonance imaging (MRI) [33]. As 1 PSMA PET/CT study included both an mpMRI-based AI model and a PSMA PET/CT model [8], internal validation was performed across 17 cohorts (n=1567 patients) for the mpMRI models [7,8,27-32,35,36,38,39,41,42,44-46], and external validation was conducted across 4 cohorts (n=439 patients) [7,32,44,46]. Moreover, there were 11 studies that compared model performance with that of radiologists [7,8,27,28,30,32,33,35,38,45,46]. Additionally, in other PSMA PET–based studies, there were 5 internal validation cohorts (n=228 patients) [8,33,34,40,43] and 1 external validation cohort (n=30 patients) [33], with 2 studies comparing against radiologists [8,33]. The included studies were published between 2019 and 2025.

Geographically, nearly half (n=10, 47%) of the studies were conducted in Asia (China), with the remainder distributed across North America/Oceania/multinational regions (n=5, 24%; United States, n=3; Australia, n=1; and multinational, n=1) and Europe (n=6, 29%; the Netherlands, n=3; Italy, n=2; and Norway, n=1). All studies were retrospective and used pathological biopsy as the gold standard. Methodologically, 18 studies used machine learning approaches, whereas 3 studies used DL. Regarding data partitioning, 12 studies applied random splitting, 6 studies used *k*-fold cross-validation, 1 study used stratified sampling, and 1 study implemented leave-one-out cross-validation. For internal validation, the most prevalent modeling techniques were least absolute shrinkage and selection operator (LASSO; 6/20, 30%), random forest (RF; 5/20, 25%), and support vector machine (3/20, 15%). External validation cohorts predominantly used RF (2/6, 33%), support vector machine (1/6, 16%), and LASSO (1/6, 16%). Study, patient, and technical characteristics are summarized in Table 2 and Tables S4-S6 in Multimedia Appendix 1.

**Table 2.** Study and patient characteristics of the included studies.

Author	Year	Country	Study design	Imaging	Reference standard	Analysis	Patients/lesions per set			Number of EPE^a^ patients/lesions
							Training	Internal validation	External validation	
Moroianu et al [27]	2022	USA	Retro^b^	mpMRI^c^	Pathology	PB^d^	74	49	—^e^	Training: 28 and internal validation: 10
Losnegard et al [28]	2020	Norway	Retro	mpMRI	Pathology	PB	228	228	—	Training: 86 and internal validation: 86
Gumus et al [29]	2025	USA	Retro	mpMRI	Pathology	LB^f^	25	25	—	Training: 12 and internal validation: 12
Priester et al [30]	2024	USA	Retro	mpMRI	Pathology	PB	142	147	—	Training: 63 and internal validation: 65
He et al [31]	2021	China	Retro	mpMRI	Pathology	LB	323	136	—	Training: 273 and internal validation: 81
Simon et al [38]	2024	Multiple countries	Retro	mpMRI	Pathology	PB	507	127	—	Training: 108 and internal validation: 27
Bai et al [44]	2021	China	Retro	mpMRI	Pathology	PB	158	68	58	Training: 60, internal validation: 23, and external validation: 25
Cuocolo et al [46]	2021	Italy	Retro	mpMRI	Pathology	PB	104	—	89	Training: 37 and external validation: 8
Damascelli et al [42]	2021	Italy	Retro	mpMRI	Pathology	LB	104	62	—	Training: 62 and internal validation: 38
Ma et al [45]	2019	China	Retro	mpMRI	Pathology	PB	143	67	—	Training: 69 and internal validation: 32
Ma et al [39]	2020	China	Retro	mpMRI	Pathology	PB	148	90	–	Training: 74 and internal validation: 26
Hou et al [32]	2021	China	Retro	mpMRI	Pathology	LB	596	150	103	Training: 151, internal validation: 40, and external validation: 33
Fan et al [41]	2022	China	Retro	mpMRI	Pathology	LB	185	47	—	Training: 40 and internal validation: 10
Xu et al [36]	2020	China	Retro	mpMRI	Pathology	LB	82	33	—	Training: 35 and internal validation: 14
van den Berg et al [7]	2023	The Netherlands	Retro	mpMRI	Pathology	LB	643	162	189	Training: 236, internal validation: 60, and external validation: 71
Pan et al [8]	2024	China	Retro	mpMRI	Pathology	PB	139	58	—	Training: 63 and internal validation: 26
Yang et al [35]	2023	China	Retro	mpMRI	Pathology	PB	274	118	–	Training: 126 and internal validation: 54
Spielvogel et al [33]	2024	Austria	Retro	PET^g^-MRI^h^	Pathology	PB	61	16	30	Training: 35, internal validation: 9, and external validation: 15
Luining et al [40]	2023	The Netherlands	Retro	PET-CT^i^	Pathology	PB	96	27	—	Training: 56 and internal validation: 15
Pan et al [8]	2024	China	Retro	PET-CT	Pathology	PB	139	58	—	Training: 63 and internal validation: 26
Yao et al [34]	2022	China	Retro	PET-CT	Pathology	PB	122	51	—	Training: 63 and internal validation: 25
Cysouw et al [43]	2021	The Netherlands	Retro	PET-CT	Pathology	PB	76	76	—	Training: 36 and internal validation: 36

a EPE: extraprostatic extension.

b Retro: retrospective.

c mpMRI: multiparametric magnetic resonance imaging.

d PB: patient based.

e Not available.

f LB: lesion based.

g PET: positron emission tomography.

h MRI: magnetic resonance imaging.

i PET-CT: positron emission tomography–computed tomography.

### Quality Assessment

**Figure 2.**
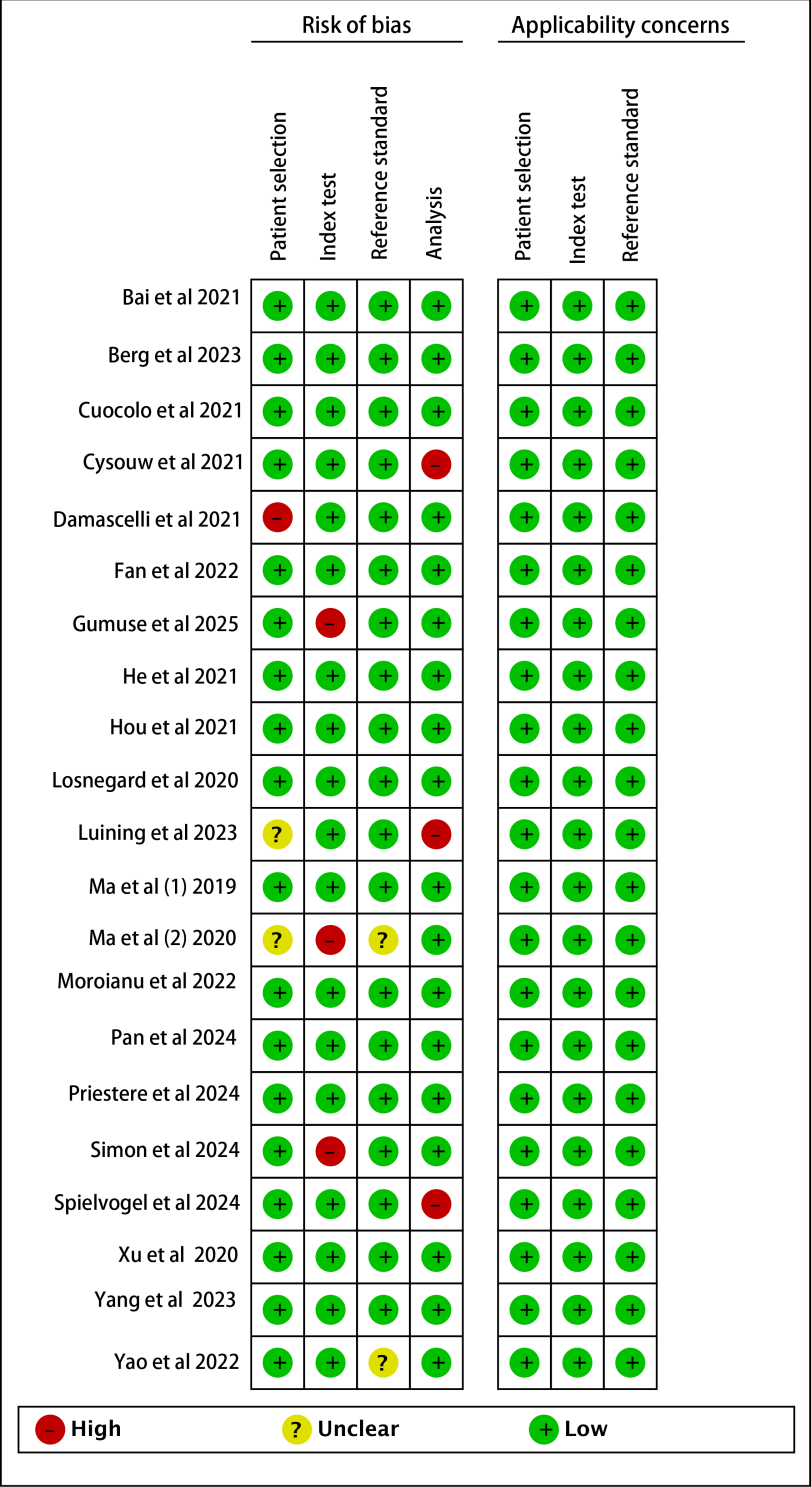
Risk of bias and applicability concerns of the included studies [7,8,27-36,38-46] using the modified quality assessment of diagnostic performance studies Quality Assessment of Diagnostic Performance Studies-2 (QUADAS-2) tool.

Risk of bias was assessed using the revised QUADAS-2 tool (Figure 2 and Table S2 in Multimedia Appendix 1). In the patient selection domain, 8 studies were rated high risk due to inappropriate exclusions. For index tests, 2 studies were deemed high risk for reporting only model names without critical training details. Three studies received unclear ratings in the reference standard domain because pathological verification might have been influenced by AI predictions. In the analysis domain, 3 studies were classified as high risk for excluding specific participants or subgroups. No unclear or high-risk concerns were identified in applicability assessments. Overall, the majority of studies demonstrated low risk, indicating acceptable methodological quality. For certainty assessment per GRADE criteria, all evidence was rated as very low (Table S3 in Multimedia Appendix 1), reflecting limited confidence in the estimated effects.

### AI Versus Radiologists: Patient-Based Performance in Internal mpMRI Validation Cohorts

**Figure 3.**
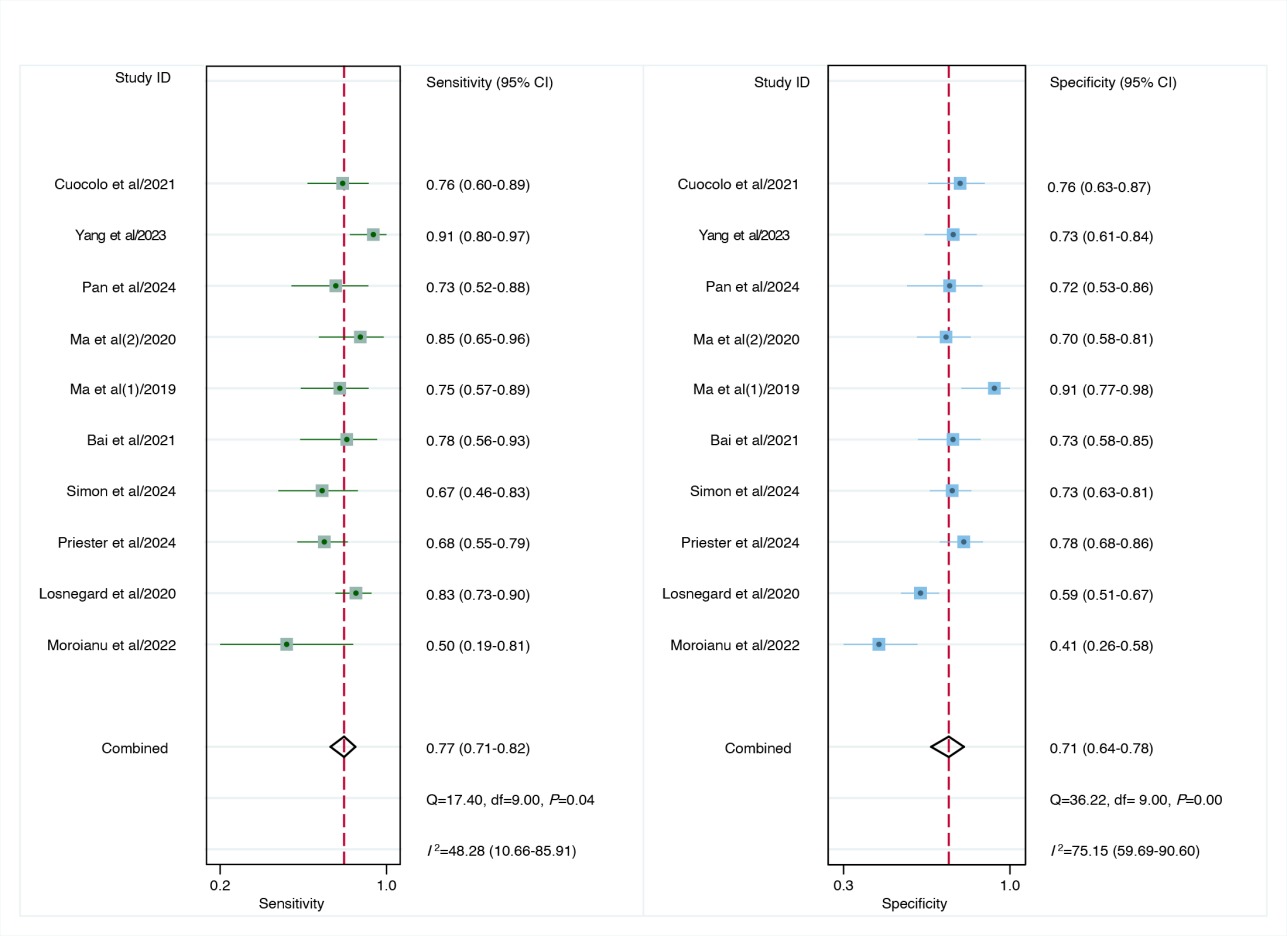
Forest plot of artificial intelligence internal validation sets of patient-based analysis for predicting preoperative extraprostatic extension in prostate cancer [8,27,28,30,35,38,39,44-46]. Squares denote the sensitivity and specificity in each study, whereas horizontal bars indicate the 95% CI.

**Figure 4.**
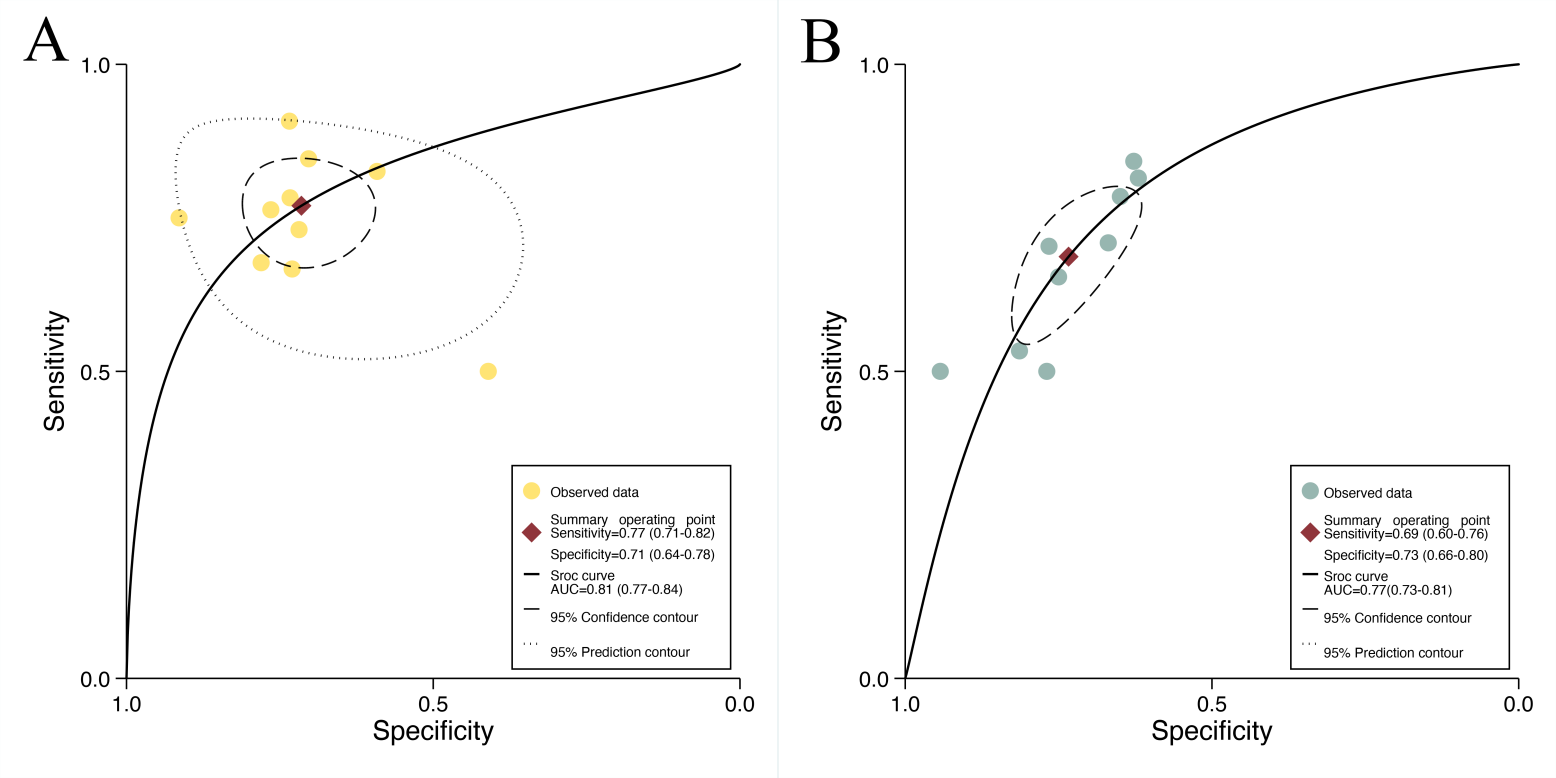
Summary receiver operating characteristic (SROC) curves for diagnosing preoperative extraprostatic extension in prostate cancer metastasis: (A) performance of patient-based artificial intelligence on the internal validation set and (B) performance of multiparametric magnetic resonance imaging assessed by radiologists. AUC: area under the curve.

**Figure 5.**
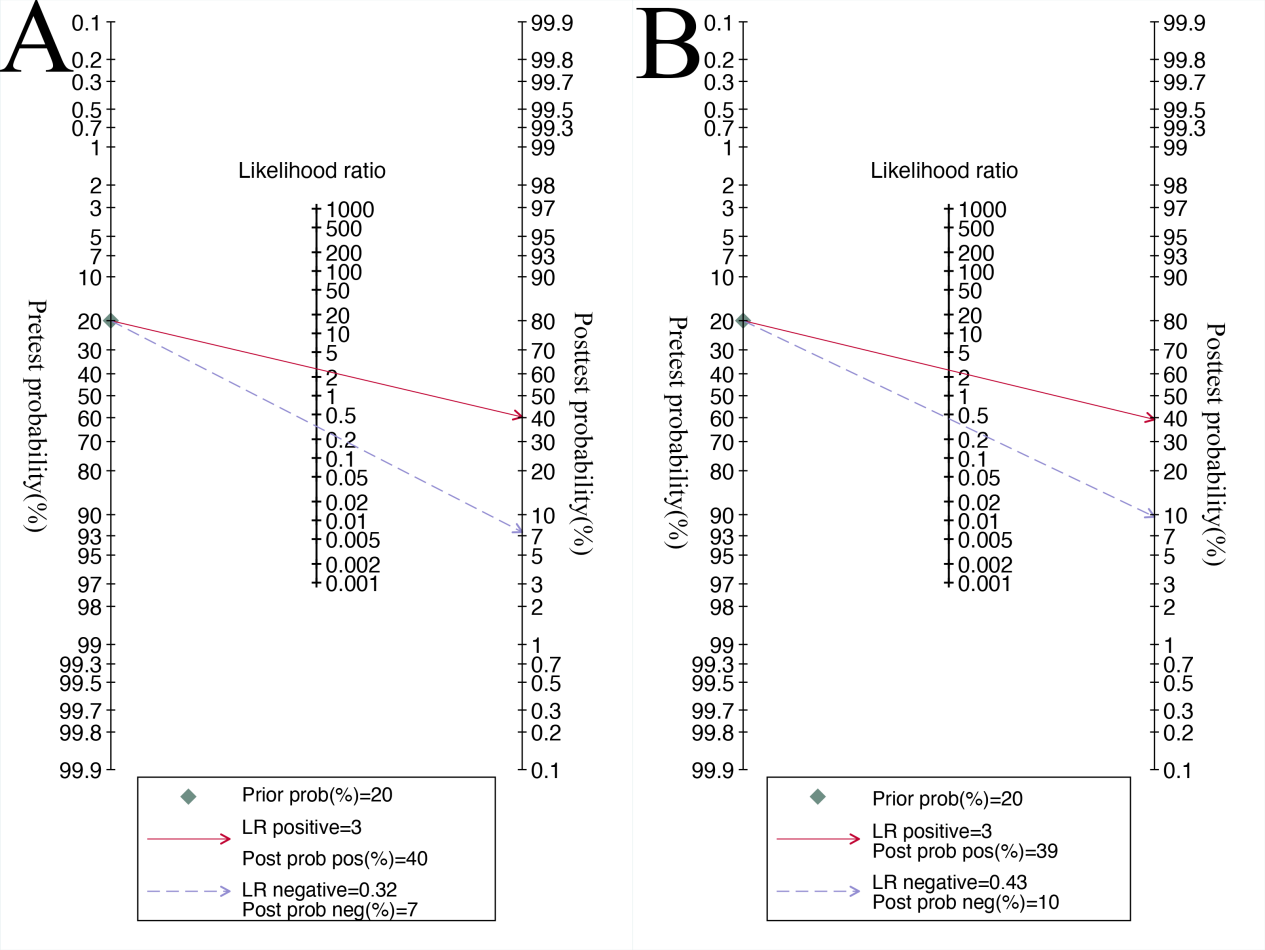
Fagan nomogram for patient-based analysis (A) artificial intelligence in predicting preoperative extraprostatic extension in prostate cancer compared to radiologists (B). LR: likelihood ratio.

**Figure 6.**
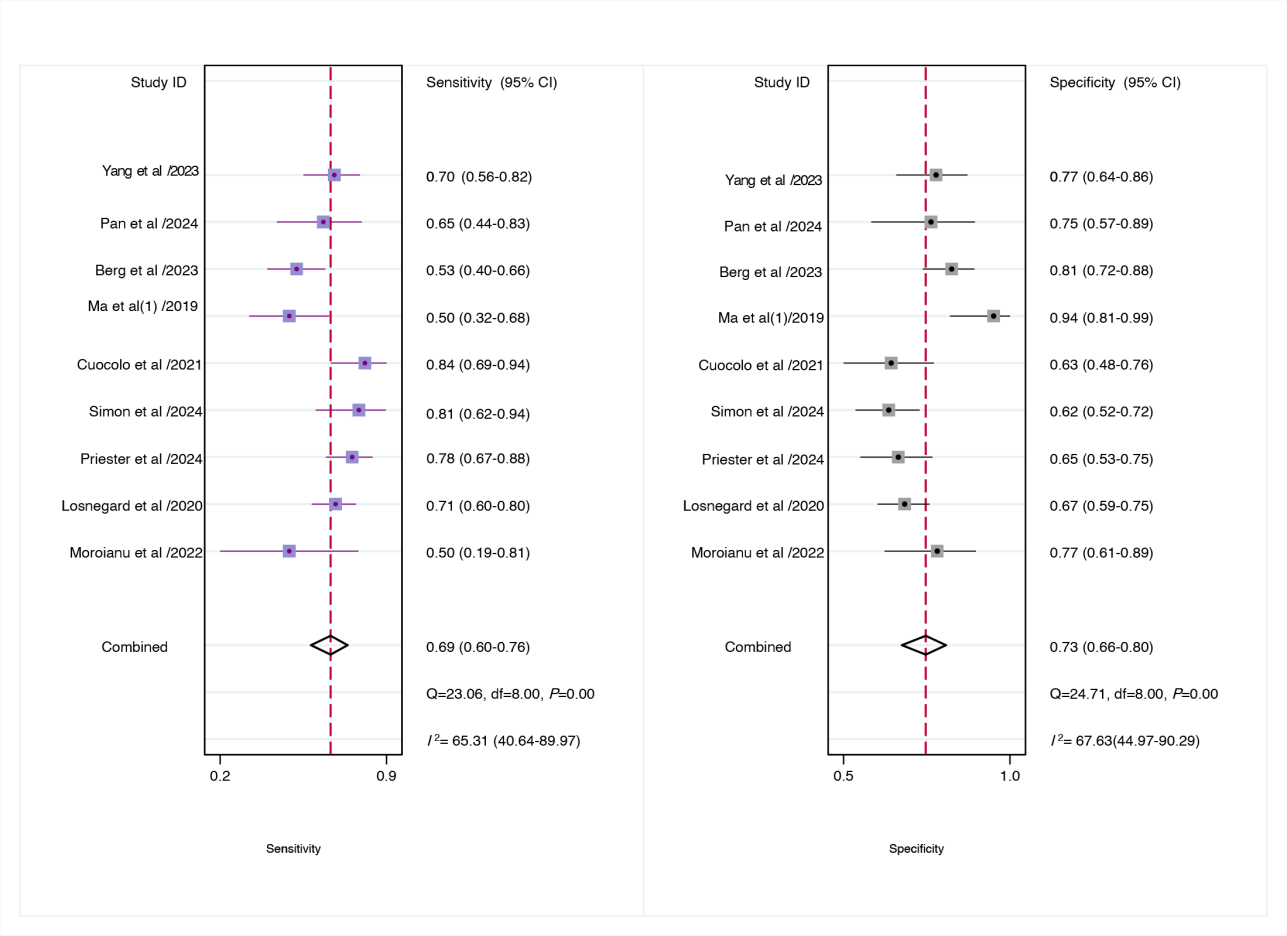
Forest plot of radiologists for predicting preoperative extraprostatic extension in prostate cancer in patient-based analysis [7,8,27,28,30,35,38,45,46]. Squares denote the sensitivity and specificity in each study, whereas horizontal bars indicate the 95% CI. df: degrees of freedom.

For internal validation sets of patient-based analysis, the pooled sensitivity of the AI model in detecting preoperative EPE of PCa was 0.77 (95% CI 0.71‐0.82), and the specificity was 0.71 (95% CI 0.64‐0.78; Figure 3), with an AUC of 0.81 (95% CI 0.77‐0.84; Figure 4A). Additionally, the meta-analysis yielded a positive likelihood ratio (PLR+) of 3 and a negative likelihood ratio (NLR−) of 0.29. Using a pretest probability of 20%, the Fagan nomogram demonstrated a posttest probability of 40% for a positive result and 7% for a negative result (Figure 5A). Moreover, for radiologists involved in patient-based analysis, the pooled sensitivity in detecting EPE was 0.69 (95% CI 0.60‐0.76), and the specificity was 0.73 (95% CI 0.66‐0.78; Figure 6), with an AUC of 0.77 (95% CI 0.73‐0.80; Figure 4B). The meta-analysis yielded a PLR+ of 3 and an NLR− of 0.43. Using a pretest probability of 20%, the Fagan nomogram demonstrated a posttest probability of 39% for a positive result and 10% for a negative result (Figure 5B).

For internal validation sets, there was no statistically significant difference in sensitivity, specificity, and AUC between the patient-based analysis AI model and radiologists (*z*=1.61, 0.43, and 1.58; *P*=.10, .67, and .11, respectively; Table 3).

**Table 3.** Comparison of diagnostic performance in patient-based analysis: mpMRI^a^-based AI^b^ versus PSMA PET^c^–based AI versus radiologists for predicting preoperative extraprostatic extension in prostate cancer.

Subgroup	Datasets, n	Sensitivity (95% CI)	Subgroup difference^d^		Specificity (95% CI)	Subgroup difference^d^		AUC^e^ (95% CI)	Subgroup difference^d^	
			*z* value	*P* value		*z* value	*P* value		*z* value	*P* value
Patient-based analysis			1.61	.10		0.43	.67		1.58	.11
mpMRI-based AI	10	0.77 (0.71‐0.82)			0.71 (0.64‐0.78)			0.81 (0.77‐0.84)		
Patient-based radiologists	9	0.69 (0.60‐0.76)			0.73 (0.66‐0.78)			0.77 (0.73‐0.80)		
Patient-based analysis			1.06	.28		0.06	.95		—^f^	—
PSMA PET–based AI	5	0.73 (0.65‐0.80)			0.61 (0.30‐0.85)			0.74 (0.70‐0.77)		
PSMA PET radiologists	2	0.73 (0.61‐0.82)			0.62 (0.45‐0.77)			—		
Patient-based analysis			0.84	.40		0.69	.49		2.77	.01
mpMRI-based AI	10	0.77 (0.71‐0.82)			0.71 (0.64‐0.78)			0.81 (0.77‐0.84)		
PSMA PET-based AI	5	0.73 (0.65‐0.80)			0.61 (0.30‐0.85)			0.74 (0.70‐0.77)		

a mpMRI: multiparametric magnetic resonance imaging.

b AI: artificial intelligence.

c PSMA PET: prostate-specific membrane antigen positron emission tomography.

d Based on 2-sided z test.

e AUC: area under the curve.

f Not applicable.

### AI Versus Radiologists: Lesion-Based Performance in Internal mpMRI Validation Cohorts

For internal validation sets of lesion-based analysis, the pooled sensitivity of the AI model in detecting preoperative EPE of PCa was 0.80 (95% CI 0.73‐0.85), and the specificity was 0.79 (95% CI 0.71‐0.86; Supplementary Figure 1 in Multimedia Appendix 1), with an area under the AUC of 0.86 (95% CI 0.83‐0.89; Figure S2 in Multimedia Appendix 1).

### Meta-Regression and Subgroup Analysis of the AI Model in mpMRI-Based Internal Validation Cohorts

The results of the meta-regression analysis indicated that the heterogeneity was primarily due to differences in region (sensitivity *P*<.001 and specificity *P*<.001), analysis type (sensitivity *P*<.001), AI model (sensitivity *P*=.01 and specificity *P*=.02), mpMRI strength (sensitivity *P*<.001), and ROI (sensitivity *P*<.001 and specificity *P*<.001; Table 4).

**Table 4.** Subgroup analysis and meta-regression analysis of mpMRI^a^-based artificial intelligence performance in internal validation sets for EPE^b^.

Subgroup	Studies, n	Sensitivity (95% CI)	Meta-regression, *P* value	Specificity (95% CI)	Meta-regression, *P* value
Region			<.001		<.001
Asia	7	0.75 (0.68-0.82)		0.72 (0.63-0.80)	
Non-Asia	9	0.82 (0.76-0.87)		0.79 (0.73-0.86)	
Analysis			<.001		.09
Lesion based	7	0.80 (0.73-0.87)		0.82 (0.76-0.89)	
Patient based	9	0.77 (0.71-0.83)		0.71 (0.64-0.77)	
AI^c^ method			.29		.36
Deep learning	3	0.68 (0.57-0.79)		0.69 (0.55-0.83)	
Machine learning	13	0.81 (0.77-0.85)		0.78 (0.72-0.84)	
AI model			.01		.02
Radiomic	8	0.76 (0.68-0.84)		0.73 (0.65-0.82)	
Radiomic and clinical	8	0.80 (0.74-0.86)		0.78 (0.71-0.85)	
Data splitting method			.19		.11
Random split	11	0.78 (0.72-0.84)		0.75 (0.69-0.82)	
K-fold cross-validation	2	0.86 (0.78-0.95)		0.70 (0.52-0.87)	
MRI^d^ strength			<.001		.53
1.5 T	2	0.86 (0.78-0.94)		0.70 (0.52-0.87)	
3 T	13	0.77 (0.72-0.83)		0.77 (0.71-0.83)	
Regions of interest			<.001		<.001
Manual delineation	11	0.79 (0.73-0.85)		0.79 (0.73-0.84)	
Automatic/semiautomatic	5	0.77 (0.69-0.86)		0.69 (0.59-0.79)	

a mpMRI: multiparametric magnetic resonance imaging.

b EPE: extraprostatic extension.

c AI: artificial intelligence.

d MRI: magnetic resonance imaging.

In terms of regional differences, sensitivity and specificity were significantly higher in non-Asian regions (sensitivity: 0.82 and specificity: 0.79) compared to Asian regions (sensitivity: 0.75 and specificity: 0.72), with significant differences (*P*<.01). In terms of analysis methods, sensitivity based on lesions (0.80) was significantly higher than that based on patients (0.77; *P*<.01). In terms of AI models, those radiomics integrating clinical features demonstrated better performance than those relying solely on radiomic feature extraction, with sensitivities of 0.80 and 0.76 and specificities of 0.78 and 0.73, respectively (*P*<.05). In terms of mpMRI strength, sensitivity at 1.5 T (0.86) was significantly higher than at 3.0 T (0.77; *P*<.01). In terms of ROI type, manual delineation showed higher sensitivity (0.79) and specificity (0.79) than automatic and semiautomatic methods (sensitivity: 0.77 and specificity: 0.69), with significant differences (*P*<.01 and *P*<.05, respectively).

### Performance in mpMRI External Validation Cohorts

For external validation sets, the sensitivity in detecting preoperative EPE of PCa was 0.66 (95% CI 0.43‐0.84), and the specificity was 0.80 (95% CI: 0.64‐0.90; Figure S3 in Multimedia Appendix 1), with an AUC of 0.80 (95% CI 0.77‐0.84; Figure S4 in Multimedia Appendix 1). The meta-analysis yielded a PLR+ of 3 and an NLR− of 0.43. Using a pretest probability of 20%, the Fagan nomogram demonstrates a posttest probability of 45% for a positive result and 10% for a negative result (Figure S5 in Multimedia Appendix 1).

### Patient-Based Performance in PSMA PET–Based Cohorts

In internal validation cohorts, the sensitivity of the AI model was 0.73 (95% CI 0.65‐0.80), and the specificity was 0.61 (95% CI 0.30‐0.85), with an AUC of 0.74 (95% CI 0.70‐0.77), as shown in Figure S6 in Multimedia Appendix 1 and Figure S7 in Multimedia Appendix 1. The meta-analysis yielded a PLR+ of 2 and an NLR− of 0.44. Using a pretest probability of 20%, the Fagan nomogram demonstrates a posttest probability of 32% for a positive result and 10% for a negative result (Figure S8 in Multimedia Appendix 1). The sensitivity of the radiologists was 0.73 (95% CI 0.53‐0.87), and the specificity was 0.62 (95% CI 0.45‐0.77), as demonstrated in Figure S9 in Multimedia Appendix 1. There was no statistically significant difference in sensitivity and specificity between the PSMA PET–based AI model and the radiologists (*z*=1.06 and 0.06; *P*=.28 and .95, respectively; Table 3).

Additionally, there were no statistically significant differences in sensitivity and specificity between the patient-based AI model and the PSMA PET–based AI model (*z*=0.84 and 0.69; *P*=.40 and .49, respectively), but the AUC of the patient-based AI model was significantly higher than that of the PSMA PET–based model (*z*=2.77; *P*=.01; Table 3). In external validation cohorts, the sensitivity of the AI model was 0.77 (95% CI 0.57‐0.89), and the specificity was 0.50 (95% CI 0.22‐0.78), as shown in Figure S9 in Multimedia Appendix 1.

### Subgroup Analysis of Different Algorithms of mpMRI-Based AI and PSMA PET–Based AI

Among mpMRI-based AI models, the most frequently used and diagnostically effective algorithm was LASSO, demonstrating sensitivity, specificity, and AUC values of 0.84 (95% CI 0.79‐0.88), 0.79 (95% CI 0.70‐0.86), and 0.83 (95% CI 0.79‐0.86), respectively (Table 5). In contrast, for PSMA PET–based AI approaches, the most commonly used algorithm was RF, which achieved sensitivity, specificity, and AUC values of 0.67 (95% CI 0.56‐0.76), 0.54 (95% CI 0.20‐0.84), and 0.74 (95% CI 0.63‐0.84), respectively.

**Table 5.** Subgroup analysis based on different AI^a^ algorithm techniques.

AI algorithms	mpMRI^b^-based AI				PSMA PET^c^–based AI			
	Studies, n	Sensitivity (95% CI)	Specificity (95% CI)	AUC^d^ (95% CI)	Studies, n	Sensitivity (95% CI)	Specificity (95% CI)	AUC (95% CI)
LR^e^	1	0.73 (0.52‐0.88)	0.72 (0.53‐0.86)	—^f^	1	0.85 (0.65‐0.96)	0.44 (0.26‐0.62)	—
RF^g^	4	0.76 (0.68‐0.82)	0.73 (0.59‐0.84)	0.81 (0.77‐0.84)	3	0.67 (0.56‐0.76)	0.54 (0.20‐0.84)	0.74 (0.63‐0.84)
SVM^h^	3	0.83 (0.73‐0.88)	0.77 (0.62‐0.88)	0.82 (0.73‐0.92)	1	0.76 (0.55‐0.91)	0.54 (0.33‐0.73)	—
EBM^i^	0	—	—	—	2	0.88 (0.67‐0.96)	0.69 (0.20‐0.95)	—
MLP^j^	1	0.75 (0.43‐0.95)	0.77 (0.46‐0.95)	—	—	—	—	—
CNN^k^	4	0.61 (0.52‐0.70)	0.71 (0.56‐0.82)	0.71 (0.67‐0.75)	—	—	—	—
LASSO^l^	7	0.84 (0.79‐0.88)	0.79 (0.70‐0.86)	0.83 (0.79‐0.86)	—	—	—	—

a AI: artificial intelligence.

b mpMRI: multiparametric magnetic resonance imaging.

c PSMA PET: prostate-specific membrane antigen positron emission tomography.

d AUC: area under the curve.

e LR: logistic regression.

f Not applicable.

g RF: random forest.

h SVM: support vector machine.

i EBM: explainable boosting machine.

j MLP: multilayer perceptron.

k CNN: convolutional neural network.

l LASSO: least absolute shrinkage and selection operator.

### Publication Bias

**Figure 7.**
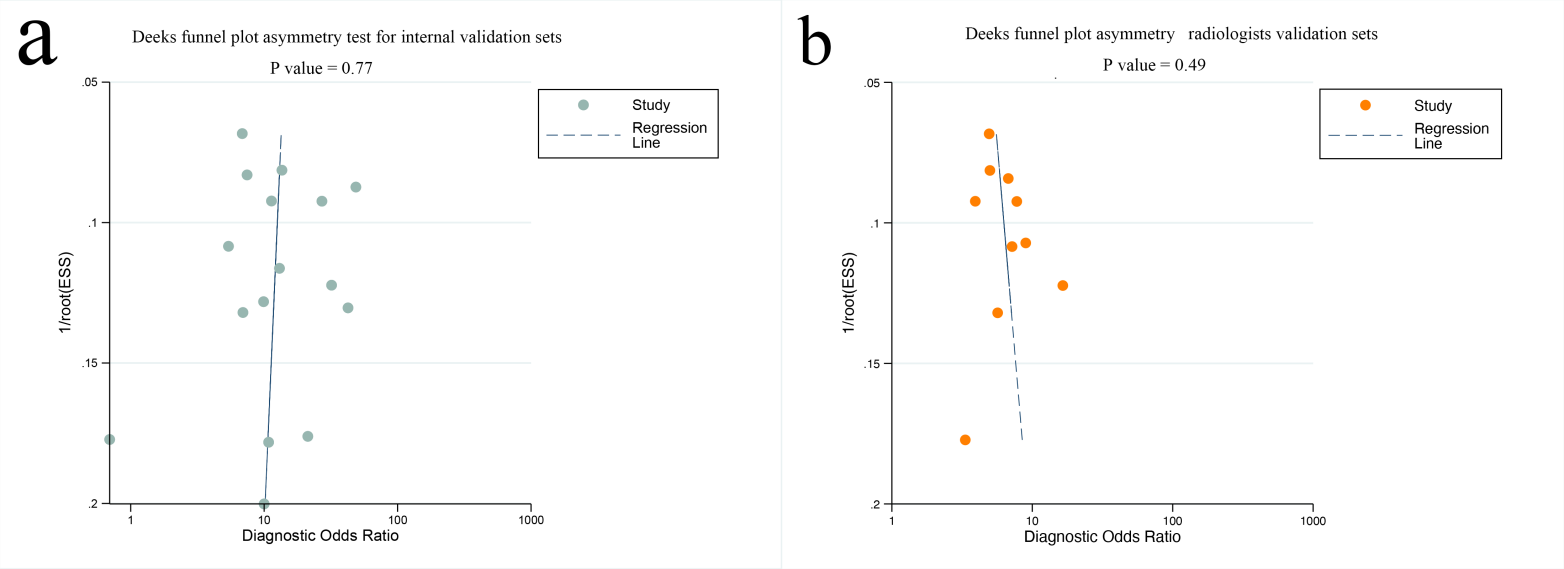
Deek funnel plot was used to evaluate the publication bias of mpMRI-based artificial intelligence (A) and radiologists (B). *P*<.05 was considered significant. ESS: effective sample size; mpMRI: multiparametric magnetic resonance imaging.

Deeks funnel plot asymmetry test found no small study effects for mpMRI-based AI without internal validation (*P*=.77) or for radiologists (*P*=.49; Figure 7A and B). No significant publication bias was observed for external validation sets (*P*=.24; Figure S10 in Multimedia Appendix 1). Additionally, there was no publication bias detected in the PSMA PET–based AI (*P*=.99; Figure S11 in Multimedia Appendix 1).

## Discussion

### Principal Findings

In this meta-analysis, AI based on mpMRI demonstrated higher sensitivity and AUC values compared to mpMRI radiologists; however, the sensitivity and specificity of PSMA PET–based AI showed no significant difference compared to PSMA PET radiologists. These findings suggest that mpMRI-based AI may have the potential to achieve higher diagnostic performance compared to radiologists, while PSMA PET–based AI appears to perform similarly to radiologists in terms of sensitivity and specificity, without demonstrating a clear advantage at this stage. From the perspective of data dimensionality and complexity, mpMRI integrates multiple sequences (such as T2-weighted imaging, diffusion-weighted imaging, and dynamic contrast-enhanced T1-weighted imaging), providing multidimensional information, including structure, cell density, and vascular perfusion. However, human radiologists face cognitive bottlenecks and subjective variability when integrating and analyzing such high-dimensional, nonlinear, and complex data [47]. For example, Hou et al [32] used an mpMRI-based AI tool (ResNeXt, a multipath residual network) for EPE detection. This tool achieved AUCs of 0.85, 0.80, and 0.72 in internal and external validation datasets, significantly outperforming mpMRI radiologists whose AUCs ranged from 0.63 to 0.74. Furthermore, Moroianu et al [27] also showed that mpMRI-based AI diagnostic performance significantly surpasses that of radiologists. In 2022, they proposed an automated detection method named Extraprostatic Extension Network based on relevant signature networks for EPE detection in PCa. Extraprostatic Extension Network achieved 0.80 sensitivity at the patient level, compared to only 0.50 sensitivity for radiologists. It is worth noting that the number of studies comparing PET-based AI to PET radiologists remains limited, which may affect the level of evidence for current results. Future research should consider larger-scale, multicenter studies.

Interestingly, our results showed that the AUC values of mpMRI-based AI are significantly higher than those of PSMA PET-based AI. This may be attributed to mpMRI’s ability to provide higher resolution images and more detailed tissue feature analysis. DL algorithms based on mpMRI, such as ResNeXt and other convolutional neural networks, demonstrate powerful feature extraction capabilities when analyzing images of patients with PCa [27,32]. Relevant studies indicate that mpMRI can better capture tumor biomarkers and information on EPE, thereby improving detection rates (accuracy) and enhancing sensitivity in clinical decision-making [38,48]. Additionally, the limitations of PSMA imaging lie in its constraints in assessing changes in the tumor microenvironment and accurately localizing tumor invasiveness, resulting in relatively lower diagnostic effectiveness of PSMA-based AI [15]. Therefore, this suggests that optimizing mpMRI image analysis combined with AI techniques can significantly enhance the application of AI in predicting PCa outcomes.

In terms of the generalizability and applicability of AI models, our results indicate that the sensitivity and AUC values of the mpMRI-based internal validation set are higher than those of the external validation set. At the same time, the specificity of the PSMA PET–based internal validation set is also higher than that of the external validation set. This phenomenon may be related to differences in the application of mpMRI and PET across various institutions and environments, where the external validation set could be influenced by factors, such as data heterogeneity, differences in image quality, and the equipment and technical standards of different hospitals [49]. Current research generally shows that AI algorithms are often trained on relatively uniform internal datasets, which may lead to performance degradation during external applications. This serves as a reminder that future research should focus on the generalizability of models, using diverse samples and standardized data processing when training models [50].

We also found that for mpMRI-based AI models, the sensitivity of the radiomics combined with the clinical model was significantly higher than that of the pure radiomics model (*P*=.01). This phenomenon may stem from the integration of radiomics data with clinical features, which enhances the model’s overall judgment ability. A stand-alone radiomics model might only use imaging features for predictions, whereas the inclusion of a clinical model allows for consideration of the patient’s physiological characteristics, medical history, and other relevant clinical data, thus achieving better performance in the comprehensive assessment of diseases [15]. Furthermore, our subgroup analysis revealed that AI models based on 1.5 T MRI exhibited significantly higher sensitivity than those using 3.0 T MRI (*P*<.001). This finding appears counterintuitive, given the technical advantages of 3.0 T systems. However, this result should be interpreted with caution, as it may be influenced by the limited number of studies in the 1.5 T subgroup (n=2) rather than reflecting a true diagnostic superiority. Additionally, we observed a numerically higher specificity for 3.0 T models (0.77 vs 0.70), suggesting a potential performance trade-off. Future studies with larger and more balanced sample sizes are warranted to validate and clarify the relationship between magnetic field strength and AI model performance in this context.

### Comparison to Prior Work

In comparing our meta-analysis with previous studies, we found the 2024 meta-analysis by Wen et al [12] reported a sensitivity of 0.77, specificity of 0.75, and an AUC of 0.88 for mpMRI-based radiomics models. In contrast, our findings yielded a sensitivity of 0.78, specificity of 0.76, and an AUC of 0.84. While our results are similar, an additional contribution of our study is that we further divided the AI dataset into internal and external validation sets to assess the generalizability of the AI models. Moreover, we compared the performance of AI with that of radiologists to evaluate the potential additive value of the AI model. Compared to the study by Wen et al, we also included an assessment of the diagnostic performance of PSMA PET–based AI in this field.

Similarly, a meta-analysis conducted by Ponsiglione et al [15] in 2024 reported a combined AUC of 0.80 for mpMRI radiomics in the detection of EPE in PCa, without providing detailed sensitivity or specificity metrics. In contrast, our findings indicated an AUC of 0.84 for mpMRI radiomics, with an AUC of 0.80 in the external validation cohort. A significant contribution of our study is the inclusion of additional sensitivity and specificity metrics, which are critical indicators of diagnostic performance. Furthermore, we conducted a multidimensional assessment of these metrics by comparing the performance of AI models against that of radiologists, as well as between mpMRI-based AI and PSMA PET–based AI.

### Heterogeneity

However, it is important to note that the substantial heterogeneity among the included studies may impact the overall sensitivity and specificity of mpMRI-based AI in the internal validation datasets. We explored the sources of heterogeneity through subgroup analysis and meta-regression, ultimately identifying region, analysis methods, AI models, the handling of ROI, and mpMRI strength as potential factors contributing to heterogeneity. Moreover, due to the limited number of studies, we were unable to investigate the sources of heterogeneity for PSMA PET–based AI, highlighting the need for further research to delve deeper into how different variables affect the diagnostic performance of PSMA PET–based assessments. Other factors include the lack of predefined subgroup analyses due to data availability, encompassing but not limited to patient age, varying severity stages of PCa, preprocessing methods, classification algorithms, sample sizes, feature selection, hyperparameter tuning, and modeling algorithms used in AI models. The combined influence of these factors may contribute to discrepancies in research outcomes, highlighting the need for future studies to further standardize and normalize the training and validation of AI models.

### Future Directions

Despite this heterogeneity, the findings indicated that AI shows significant potential in predicting preoperative EPE in PCa, possibly even surpassing the capabilities of radiologists and PSMA PET–based AI. AI has the potential to alleviate the workload of clinical practitioners, reduce the incidence of misdiagnoses and missed diagnoses, and prevent adverse outcomes associated with the disease [4].

Furthermore, AI developed in different countries demonstrates promise in enhancing screening efficiency, particularly in resource-constrained or remote areas where access to specialized expertise is limited [51]. These advantages may support future health policy directions aimed at improving diagnostic equity and optimizing radiological resources, especially in underserved regions. Future implementation of AI-based mpMRI in primary health care systems, such as family medicine, could facilitate the early detection and timely management of EPE in PCa [52]. Additionally, AI can provide preliminary interpretations of imaging examinations, allowing clinicians to process cases more rapidly, improve turnaround times, and enhance access to specialist-level reports, ultimately alleviating pressure on the health care system [53]. Our findings may also inform future regulatory policies regarding the clinical integration of AI technologies, including standards for validation, deployment, and reimbursement. Moreover, future studies should incorporate decision-curve analysis to evaluate the clinical net benefit of AI-assisted strategies across a range of clinically reasonable probability thresholds for EPE, which would further clarify its utility in supporting clinical decision-making.

Although our study indicates that mpMRI-based AI demonstrates high diagnostic performance, further verification is needed concerning mpMRI-based AI versus PSMA PET–based AI due to the limited number of PSMA PET samples. Furthermore, future AI models based solely on mpMRI imaging may encounter limitations, as they fail to comprehensively analyze patients undergoing different radiological imaging assessments [54]. For instance, patients may receive both mpMRI and PSMA PET scans simultaneously. Therefore, future AI models may need to be trained across multiple imaging modalities and correlate findings with the clinical context of patients, thereby providing more comprehensive insights through reports [55]. This need highlights a significant gap that future research should prioritize addressing.

### Limitations

Several limitations of this meta-analysis should be considered when interpreting the results. First, all included studies were retrospective in design, which may introduce potential selection bias. Therefore, well-designed prospective studies are needed to validate our findings. Second, due to potential patient overlap across studies, we only extracted the best-performing algorithms from each article, which might lead to overoptimistic results. Future studies are necessary to compare the diagnostic performance across different specific algorithms. Third, as some original studies did not provide complete diagnostic contingency tables, we reconstructed the data by estimating the Youden index from ROC curve points to derive TP, FP, FN, and TN values. This process of data reconstruction may introduce estimation errors. Fourth, to ensure feasibility in literature screening and data extraction, we only included articles published in English, which might introduce language bias by excluding relevant studies in other languages. Fifth, the number of external validation cohorts available for analysis remains limited (a total of 6 cohorts), which restricts the robustness of assessing model generalizability. More high-quality external validation studies are needed to verify the performance of these AI models in real-world clinical settings. Finally, due to data limitations, we were unable to stratify radiologists by experience level, highlighting the need for further research to evaluate differences in diagnostic performance between radiologists of varying expertise and AI algorithms.

### Conclusions

mpMRI-based AI demonstrated improved diagnostic performance for preoperative prediction of EPE in PCa compared to conventional radiological assessment, achieving a higher AUC. However, PSMA PET–based AI models currently offer no significant advantage over either mpMRI AI or radiologists. Limitations include the retrospective design and high heterogeneity, which may introduce bias and affect generalizability. Larger, diverse cohorts are essential for confirming these findings and optimizing the integration of AI in clinical practice.

## Supplementary material

10.2196/80981 Multimedia Appendix 1 Search methods, quality assessments, technical details, and artificial intelligence diagnostic performance analyses.

10.2196/80981 Checklist 1 PRISMA-DTA checklist.

## Abbreviations

AI: artificial intelligence
AUC: area under the curve
CT: computed tomography
DL: deep learning
EPE: extraprostatic extension
FN: false negative
FP: false positive
GRADE: Grading of Recommendations, Assessment, Development, and Evaluations
LASSO: least absolute shrinkage and selection operator
MeSH: Medical Subject Headings
mpMRI: multiparametric magnetic resonance imaging
MRI: magnetic resonance imaging
NLR: negative likelihood ratio
PCa: prostate cancer
PITROS: Participants, Index Test, Target Conditions, Reference Standard, Outcomes, Settings
PLR: positive likelihood ratio
PRISMA: Preferred Reporting Items for Systematic Reviews and Meta-Analysis
PRISMA-DTA: Reporting Items for Systematic Reviews and Meta-Analyses of Diagnostic Test Accuracy
PSMA PET: prostate-specific membrane antigen positron emission tomography
QUADAS-2: Quality Assessment of Diagnostic Performance Studies
RF: random forest
ROC: receiver operating characteristic curve
ROI: region of interest
TN: true negative
TP: true positive
