# Supplementary material for: Artificial Intelligence–Enabled Imaging for Predicting Preoperative Extraprostatic Extension in Prostate Cancer: Systematic Review and Meta-Analysis
Source: J Med Internet Res. 2025 Dec 9;27:e80981. doi: 10.2196/80981 (PMC12688051; doi:10.2196/80981)

Table S1 Search strategy in PubMed, Embase and Web of Science.

| Database | Search strategy |
| --- | --- |
| PubMed | ("Radiomics"[Mesh] OR "Artificial Intelligence"[Mesh] OR "Machine Learning"[Mesh] OR "Deep Learning"[Mesh] OR "Artificial Intelligence"[Title/Abstract] OR "AI"[Title/Abstract] OR "Machine Learning"[Title/Abstract] OR "Deep Learning"[Title/Abstract] OR “Machine Intelligence”[Title/Abstract] OR “Radiomic”[Title/Abstract]) AND ("Prostatic Neoplasms"[Mesh] OR "Prostate Cancer"[Title/Abstract] OR "Prostatic Cancer"[Title/Abstract] OR "Prostate Carcinoma"[Title/Abstract] OR "Prostatic Carcinoma"[Title/Abstract] OR "Prostate Neoplasm*"[Title/Abstract]) AND ("Neoplasm Invasiveness"[Mesh] OR "Extraprostatic Extension"[Title/Abstract] OR "EPE"[Title/Abstract] OR "Extracapsular Extension"[Title/Abstract] OR "Capsular Penetration"[Title/Abstract] OR "Prostate Cancer Extension"[Title/Abstract] OR "Prostate Cancer Invasion"[Title/Abstract]) |
| Embase | ('Radiomics'/exp OR 'Artificial Intelligence'/exp OR 'Machine Learning'/exp OR 'Deep Learning'/exp OR 'Artificial Intelligence':ab,ti OR 'AI':ab,ti OR 'Machine Learning':ab,ti OR 'Deep Learning':ab,ti OR 'Machine Intelligence':ab,ti OR 'Radiomic':ab,ti) AND ('Prostate Tumor'/exp OR 'Prostate Cancer':ab,ti OR 'Prostatic Cancer':ab,ti OR 'Prostate Carcinoma':ab,ti OR 'Prostatic Carcinoma':ab,ti OR 'Prostate Neoplasm*':ab,ti) AND (‘Neoplasm Invasiveness’/exp OR 'Extraprostatic Extension'/exp OR 'Extraprostatic Extension':ab,ti OR 'EPE':ab,ti OR 'Extracapsular Extension':ab,ti OR 'Capsular Penetration':ab,ti OR 'Prostate Cancer Extension':ab,ti OR 'Prostate Cancer Invasion':ab,ti) |
| Web of Science | TS=("Radiomics" OR "Artificial Intelligence" OR "Machine Learning" OR "Deep Learning" OR "Machine Intelligence" OR "Radiomic") AND TS=("Prostatic Neoplasms" OR "Prostate Cancer" OR "Prostatic Cancer" OR "Prostate Carcinoma" OR "Prostatic Carcinoma" OR "Prostate Neoplasm*") AND TS=(“Neoplasm Invasiveness” OR "Extraprostatic Extension" OR "EPE" OR "Extracapsular Extension" OR "Capsular Penetration" OR "Prostate Cancer Extension" OR "Prostate Cancer Invasion") |

Table S2 Modified QUADAS-2 tool for the included studies

| Author | | Risk of bias | | | | Applicability concerns | | |
| --- | --- | --- | --- | --- | --- | --- | --- | --- |
|  |  | Patient  selection ^a^ | Index  test ^b^ | Reference standard ^c^ | Analysis ^d^ | Patient selection ^e^ | Index test ^f^ | Reference standard ^g^ |
| Gumus et al. | 2025 | L | H | L | L | L | L | L |
| Pan et al. | 2024 | L | L | L | L | L | L | L |
| Spielvogel et al. | 2024 | L | L | L | H | L | L | L |
| Simon et al. | 2024 | L | H | L | L | L | L | L |
| Priester et al. | 2024 | L | L | L | L | L | L | L |
| Berg et al. | 2023 | L | L | U | L | L | L | L |
| Yang et al. | 2023 | L | L | L | L | L | L | L |
| Luining et al. | 2023 | U | L | L | H | L | L | L |
| Moroianu et al. | 2022 | L | L | L | L | L | L | L |
| Fan et al. | 2022 | L | L | L | L | L | L | L |
| Yao et al. | 2022 | L | L | U | L | L | L | L |
| Cysouw et al. | 2021 | L | L | L | H | L | L | L |
| Hou et al. | 2021 | L | L | L | L | L | L | L |
| Cuocolo et al. | 2021 | L | L | L | L | L | L | L |
| Damascelli et al. | 2021 | H | L | L | L | L | L | L |
| Bai et al. | 2021 | L | L | L | L | L | L | L |
| He et al.2021 | 2021 | L | L | L | L | L | L | L |
| Losnegard et al. | 2020 | L | L | L | L | L | L | L |
| Xu et al. | 2020 | L | L | L | L | L | L | L |
| Ma et al.(1) | 2019 | L | L | L | L | L | L | L |
| Ma et al.(2) | 2020 | U | H | U | L | L | L | L |

L low; H high; U unclear.

a. Patient selection

·Low risk: No inappropriate exclusions.

·High risk: Inappropriate exclusions (e.g., excluding patients under 18, restricting to specific treatments/subtypes/timeframes).

·Unclear: Insufficient information to assess exclusions.

b. Index test

·Low risk: Detailed model training/validation processes provided or cited from a prior publication with full modification details.

·High risk: Only model name reported without key training details (e.g., algorithm unspecified).

·Unclear: Model name given but training process indeterminable.

c. Reference standard

·Low risk: Final diagnosis made blinded to AI results.

·High risk: AI results used to inform final diagnosis.

·Unclear: Blinding status unreported.

d. Analysis

·Low risk: All enrolled participants included in meta-analysis.

·High risk: Selective exclusion of participants/subgroups.

·Unclear: Inclusion criteria inadequately described.

e. Patient selection

·Low risk: Study population aligns with meta-analysis inclusion criteria.

·High risk: Study includes ineligible patients per meta-analysis criteria.

·Unclear: Population eligibility unclear.

f. Index test

·Low risk: AI definition matches meta-analysis criteria.

·High risk: AI definition partially deviates from criteria.

·Unclear: AI definition adequacy unverifiable.

g. Reference standard

·Low risk: Reference standard aligns with meta-analysis criteria.

·High risk: Reference standard inconsistently applied.

·Unclear: Reference standard details missing.

Table S3 GRADE scoring assessments in all the pooled outcomes

| Dataset | Outcome | Risk of Bias ^a^ | Inconsistency ^b^ | Indirectness ^c^ | Imprecision ^d^ | Publication Bias^e^ | Total Downgrade | Final Rating |
| --- | --- | --- | --- | --- | --- | --- | --- | --- |
| Internal validation dataset for MRI-based AI | Sensitivity | 1 | 0 | 1 | 1 | 0 | 3 | Very low |
|  | Specificity | 1 | 0 | 1 | 1 | 0 | 3 | Very low |
| External validation dataset for MRI-based AI | Sensitivity | 0 | 1 | 1 | 1 | 0 | 3 | Very low |
|  | Specificity | 0 | 1 | 1 | 1 | 0 | 3 | Very low |
| MRI radiologist | Sensitivity | 1 | 0 | 1 | 0 | 0 | 2 | Moderate |
|  | Specificity | 1 | 0 | 1 | 0 | 0 | 2 | Moderate |
| Internal validation dataset for PSMA PET-based AI | Sensitivity | 1 | 1 | 1 | 0 | 0 | 3 | Very low |
|  | Specificity | 1 | 1 | 1 | 1 | 0 | 4 | Very low |
| External validation dataset for PSMA PET-based AI | Sensitivity | 1 | 1 | 0 | 1 | 0 | 3 | Very low |
|  | Specificity | 1 | 1 | 0 | 1 | 0 | 3 | Very low |
| PSMA PET radiologist | Sensitivity | 1 | 1 | 0 | 1 | 0 | 3 | Very low |
|  | Specificity | 1 | 1 | 0 | 1 | 0 | 3 | Very low |

a. Risk of Bias

Assessed using tools like QUADAS-2 to evaluate study design and methodology.

Downgrade by 1 level if at least one study has a high risk of bias.

b. Inconsistency

Measured using I² statistics to assess heterogeneity across studies.

Downgrade by 1 level if I² > 50% and the source of heterogeneity cannot be explained.

If the heterogeneity is identified (e.g., through meta-regression), no downgrade is applied.

c. Indirectness

Evaluates whether study populations, interventions, or outcomes differ from the target research question.

Patient Indirectness: Downgrade if the included population significantly deviates from the target population (e.g., specific subgroups).

Outcome Indirectness: Downgrade if inconsistent outcome measures are used, such as estimating sensitivity/specificity indirectly from ROC-based Youden’s Index.

d. Imprecision

Assessed based on confidence intervals (CIs) and sample size sufficiency.

For sensitivity/specificity: Downgrade if 95% CI width > 0.20 or if CIs cross clinical thresholds (e.g., 0.80).

For DOR: Downgrade if CI range exceeds 1× the lower limit or includes values < 10.

Sample size threshold: Total sample < 500 or positive/negative cases < 100.

e. Publication Bias

Evaluated using Deek’s Funnel Plot or similar tools.

Downgrade by 1 level if the funnel plot shows significant asymmetry or if *P*-value < 0.05 in Deek’s tests.

Table S4 Technical aspects of included studies

| Author | Year | AI method | AI model | Optimal AI algorithms ^a^ | Data splitting method | Internal validation sets | | | | External validation sets | | | | Radiologists | | | |
| --- | --- | --- | --- | --- | --- | --- | --- | --- | --- | --- | --- | --- | --- | --- | --- | --- | --- |
|  |  |  |  |  |  | TP | FP | FN | TN | TP | FP | FN | TN | TP | FP | FN | TN |
| Moroianu et al. | 2022 | Deep learning | Radiomic | CNN | Random split | 5 | 23 | 5 | 16 | NA | NA | NA | NA | 5 | 9 | 5 | 30 |
| Losnegard et al. | 2020 | Machine learning | Radiomic & Clinical | RF | 10-fold cross-validation | 71 | 58 | 15 | 84 | NA | NA | NA | NA | 61 | 47 | 25 | 95 |
| Gumus et al. | 2025 | Machine learning | Radiomic | MLP | Leave-one-out cross-validation | 9 | 3 | 3 | 10 | NA | NA | NA | NA | NA | NA | NA | NA |
| Priester et al. | 2024 | Deep learning | Radiomic & Clinical | CNN | NA | 44 | 18 | 21 | 64 | NA | NA | NA | NA | 51 | 27 | 14 | 50 |
| He et al. | 2021 | Machine learning | Radiomic & Clinical | LASSO | Random split | 71 | 7 | 10 | 48 | NA | NA | NA | NA | NA | NA | NA | NA |
| Simon et al. | 2024 | Machine learning | Radiomic | RF | Random split | 18 | 27 | 9 | 73 | NA | NA | NA | NA | 22 | 38 | 5 | 62 |
| Bai et al. | 2021 | Machine learning | Radiomic & Clinical | LASSO | Random split | 18 | 12 | 5 | 33 | 20 | 16 | 5 | 17 | NA | NA | NA | NA |
| Cuocolo et al. | 2021 | Machine learning | Radiomic | SVM | 10-fold cross-validation | NA | NA | NA | NA | 29 | 12 | 9 | 39 | 32 | 19 | 6 | 32 |
| Damascelli et al. | 2021 | Machine learning | Radiomic | SVM | 10-fold cross-validation | 34 | 4 | 4 | 20 | NA | NA | NA | NA | NA | NA | NA | NA |
| Ma et al.(1) | 2019 | Machine learning | Radiomic & Clinical | LASSO | Random split | 24 | 3 | 8 | 32 | NA | NA | NA | NA | 16 | 2 | 16 | 33 |
| Ma et al.(2) | 2020 | Machine learning | Radiomics | LASSO | Random split | 22 | 19 | 4 | 45 | NA | NA | NA | NA | NA | NA | NA | NA |
| Hou et al. | 2021 | Deep learning | Radiomics | CNN | Random split | 30 | 23 | 10 | 87 | 9 | 8 | 24 | 62 | 28 | 41 | 12 | 69 |
| Fan et al. | 2022 | Machine learning | Radiomic & Clinical | SVM | Random split | 8 | 10 | 2 | 27 | NA | NA | NA | NA | NA | NA | NA | NA |
| Xu et al. | 2020 | Machine learning | Radiomic | LASSO | Random split | 10 | 2 | 4 | 17 | NA | NA | NA | NA | NA | NA | NA | NA |
| Berg et al. | 2023 | Machine learning | Radiomic & Clinical | RF | Stratified sampling | 43 | 16 | 17 | 86 | 51 | 13 | 20 | 105 | 32 | 19 | 28 | 83 |
| Pan et al. | 2024 | Machine learning | Radiomic | LR | Random split | 19 | 9 | 7 | 23 | NA | NA | NA | NA | 17 | 8 | 9 | 24 |
| Yang et al. | 2023 | Machine learning | Radiomic & Clinical | LASSO | Random split | 49 | 17 | 5 | 47 | NA | NA | NA | NA | 38 | 15 | 16 | 49 |
| Spielvogel et al. | 2024 | Machine learning | Radiomic & Clinical | EBM | 100-fold stratified Monte Carlo cross-validation | 7 | 1 | 2 | 6 | 14 | 6 | 1 | 9 | 8 | 2 | 7 | 13 |
| Luining et al. | 2023 | Machine learning | Radiomic | RF | 5-fold cross-validation | 36 | 4 | 14 | 42 | 9 | 3 | 6 | 1 | NA | NA | NA | NA |
| Pan et al. | 2024 | Machine learning | Radiomic | LR | Random split | 22 | 18 | 4 | 14 | NA | NA | NA | NA | 20 | 11 | 6 | 21 |
| Yao et al. | 2022 | Machine learning | Radiomic & Clinical | SVM | Random split | 19 | 12 | 6 | 14 | NA | NA | NA | NA | NA | NA | NA | NA |
| Cysouw et al. | 2021 | Machine learning | Radiomic | RF | 5-fold cross-validation | 23 | 29 | 13 | 6 | NA | NA | NA | NA | NA | NA | NA | NA |

TP true positive; TN true negative; FP false positive; FN false positive; NA not available; CNN convolutional neural network; MLP multilayer perceptron; LASSO least absolute shrinkage and selection operator; SVM support vector machine classier; RF random forest model; BN Bayesian network; DL deep learning; LR logistic regression; EBM explainable boosting machine.

^a^ Optimal means the artificial intelligence algorithm with the highest AUC value

Table S5 Technical aspects of included studies.

| Author | Year | Scanner Modality(MRI) | MRI sequence | MRI strength | Regions of interest | Stage |
| --- | --- | --- | --- | --- | --- | --- |
| Moroianu et al. | 2022 | GE Healthcare | T2WI,DWI, ADC | 3.0 T | Automatic delineation | Pre-operation |
| Losnegard et al. | 2020 | Siemens Medical | T1WI,T2WI,DWI,DCE | 1.5 T | Semi-automaticdelineation | Pre-operation |
| Gumus et al. | 2025 | Siemens Medical | T1WI,T2WI,DWI,DCE | 3.0 T | Manual delineation | Pre-operation |
| Priester et al. | 2024 | NA | T2WI,DWI,DCE | 1.5 T, 3.0 T | Manual delineation | Pre-operation |
| He et al. | 2021 | Siemens Medical | T2WI, DWI, ADC | 3.0 T | Manual delineation | Pre-operation |
| Simon et al. | 2024 | NA | T2WI, DWI, ADC | 3.0 T | Manual delineation | Pre-operation |
| Bai et al. | 2021 | Siemens Medical, GE Healthcare | T2WI, ADC, DWI | 3.0 T | Manual delineation | Pre-operation |
| Cuocolo et al. | 2021 | Philips Medical, Siemens Medical | T2WI, DWI,ADC | 1.5 T, 3.0 T | Manual delineation | Pre-operation |
| Damascelli et al. | 2021 | Philips Medical | T2WI, ADC, DWI,DCE | 1.5 T | Semi-automaticdelineation | Pre-operation |
| Ma-1 et al. | 2019 | GE Healthcare, Philips Medical | T2WI, DWI,DCE | 3.0 T | Manual delineation | Pre-operation |
| Ma-2 et al. | 2020 | GE Healthcare, Philips Medical | T2WI, DWI,DCE | 3.0 T | Manual delineation | Pre-operation |
| Hou et al. | 2021 | Siemens Medical | T1WI, T2WI, DWI, ADC | 3.0 T | Manual delineation | Pre-operation |
| Fan et al. | 2022 | NA | T2WI, DWI, DCE | 3.0 T | Manual delineation | Pre-operation |
| Xu et al. | 2020 | GE Healthcare | DWI, ADC, T2WI,DCE | 3.0 T | Manual delineation | Pre-operation |
| Berg et al. | 2023 | Philips Medical, Siemens Medical | T2WI, ADC, DWI | 3.0 T | Semi-automaticdelineation | Pre-operation |
| Pan et al. | 2024 | Philips Medical, GE Healthcare | T2WI, ADC, DWI | 3.0 T | Semi-automaticdelineation | Pre-operation |
| Yang et al. | 2023 | Siemens Medical | T1WI, T2WI, DWI, ADC | 3.0 T | Manual delineation | Pre-operation |

MRI magnetic resonance imaging; ROI Regions of interest; T1WI T1-weighted imaging; T2WI T2-weighted imaging; DWI diffusion-weighted imaging; ADC apparent diffusion coefficient; DCE dynamic contrast enhanced

Table S6 Technical aspects of included studies

| Author | Year | Scanner Modality (PET) | Radiotracer | Ligand dose | Regions of interest (ROI) | Stage |
| --- | --- | --- | --- | --- | --- | --- |
| Spielvogel et al. | 2024 | Siemens Healthineers | 68Ga-PSMA-11 | NA | Manual delineation | Pre-operation |
| Luining et al. | 2023 | Siemens Healthineers Philips Medical | 18F-DCFPyL | 308 MBq  302 MBq | Semi-automatic | Pre-operation |
| Pan et al. | 2024 | Philips Medical | 18F-PSMA-1007 | NA | Semi-automatic | Pre-operation |
| Yao et al. | 2022 | Philips Medical | 18F-PSMA-1007 | NA | Semi-automatic | Pre-operation |
| Cysouw et al. | 2021 | Philips Medical | 18F-DCFPyL | 310.1 ± 16.2 MBq | Semi-automatic | Pre-operation |

MRI magnetic resonance imaging; ROI Regions of interest; 68Ga-PSMA-11 Gallium-68 prostate-specific membrane antigen-11; 18F-DCFPyL 18F-diacylglycerol pyridyl ligand; 18F-PSMA-1007 18F-prostate-specific membrane antigen-1007; MBq megabecquerels

Figure S1: Forest plot of artificial intelligence internal validation sets of lesion-based analysis for predicting preoperative extraprostatic extension in prostate cancer. Squares denote the sensitivity and specificity in each study, while horizontal bars indicate the 95% confidence interval.


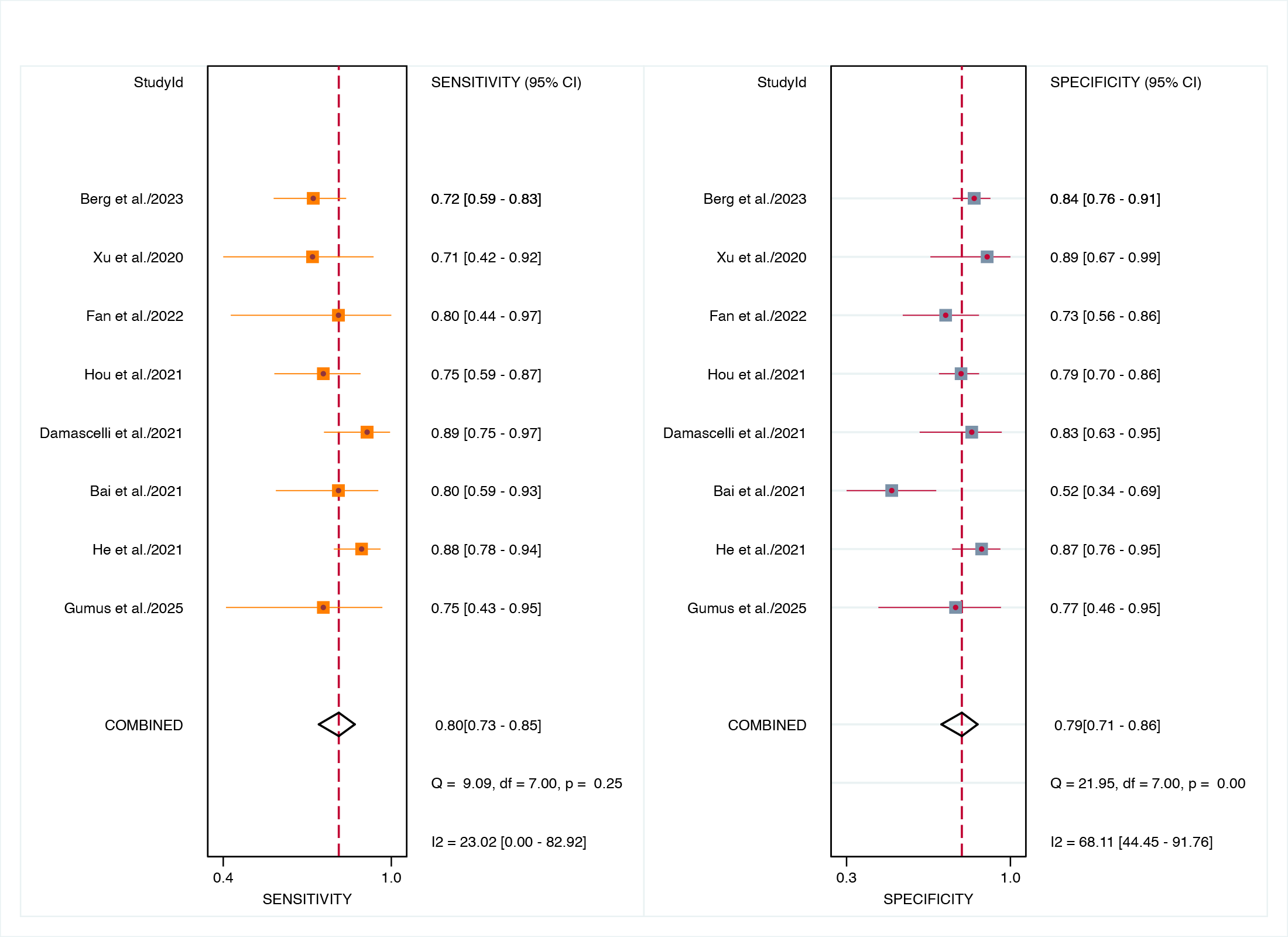


Figure S2: Summary receiver operating characteristic (SROC) curves for diagnosing preoperative extraprostatic extension in prostate cancer metastasis: performance of lesion-based artificial intelligence on the internal validation set.


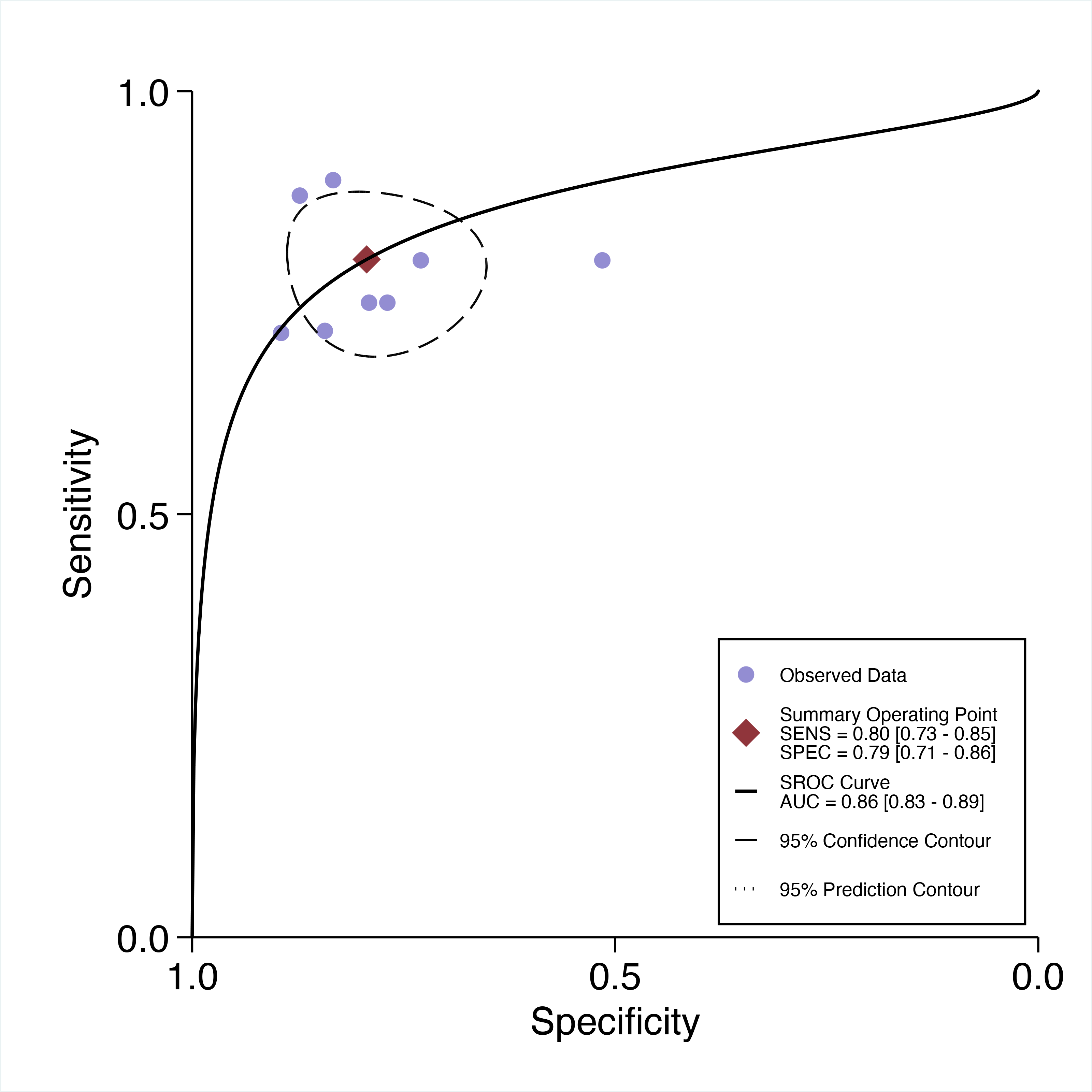


Figure S3: Forest plot of diagnostic performance of mpMRI-based artificial intelligence external validation sets for predicting preoperative extraprostatic extension in prostate cancer. The sensitivity and specificity of each study were represented by squares, with horizontal bars indicating the 95% confidence intervals.


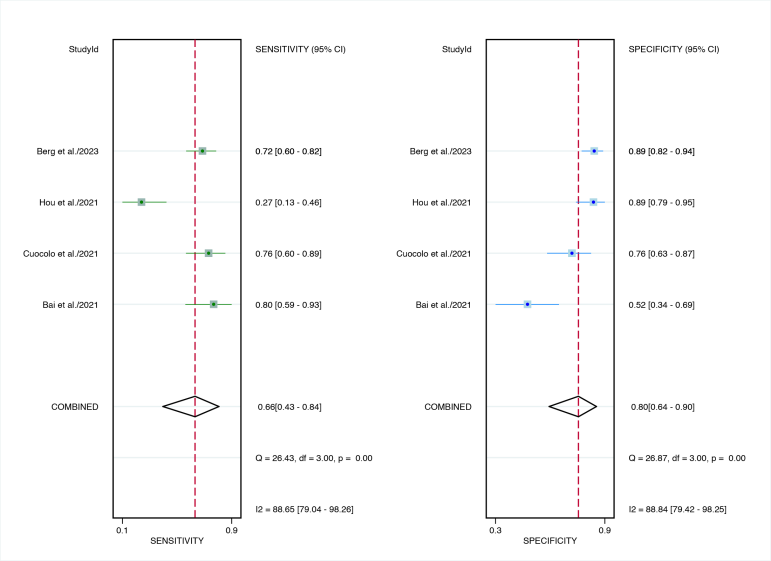


Figure S4: Summary receiver operating characteristic (SROC) curves of diagnostic performance of mpMRI based-artificial intelligence external validation sets for predicting preoperative extraprostatic extension in prostate cancer.


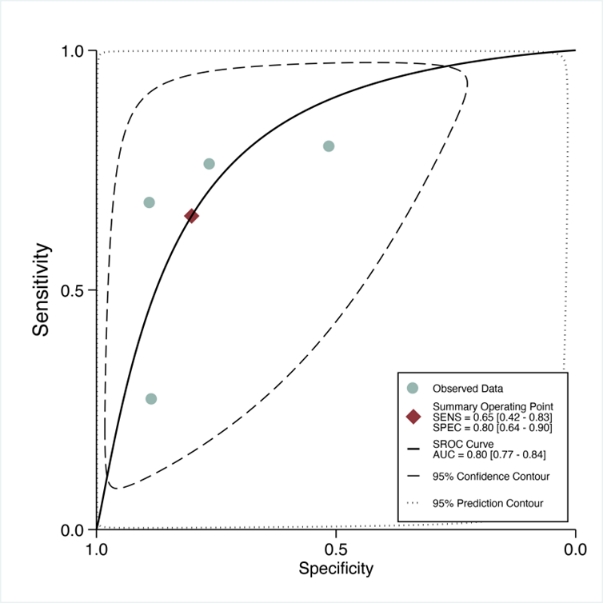


Figure S5: Fagan plot of diagnostic performance of mpMRI based-artificial intelligence external validation sets for predicting preoperative extraprostatic extension in prostate cancer.


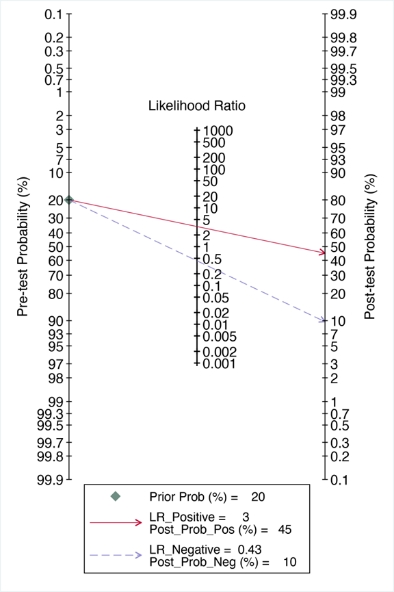


Figure S6: Forest plot of the diagnostic performance of PSMA PET-based AI internal validation sets for predicting preoperative extraprostatic extension in prostate cancer. The sensitivity and specificity of each study were represented by squares, with horizontal bars indicating the 95% confidence intervals.


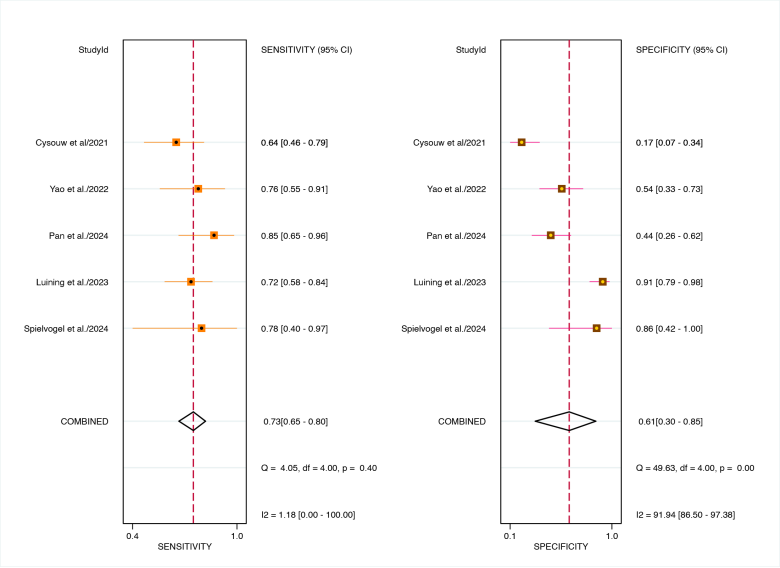


Figure S7: Summary receiver operating characteristic (SROC) curves of diagnostic performance of PSMA PET based-artificial intelligence internal validation sets for predicting preoperative extraprostatic extension in prostate cancer.


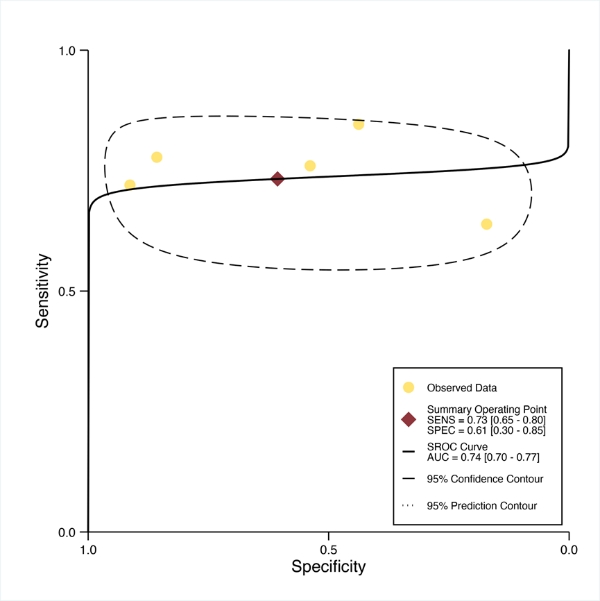


Figure S8: Fagan plot of diagnostic performance of PSMA PET based-artificial intelligence internal validation sets for predicting preoperative extraprostatic extension in prostate cancer.


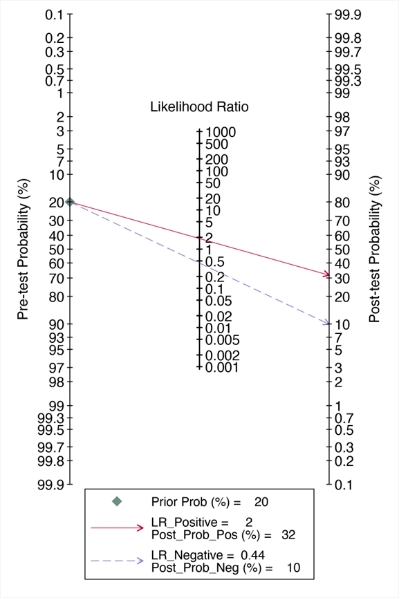


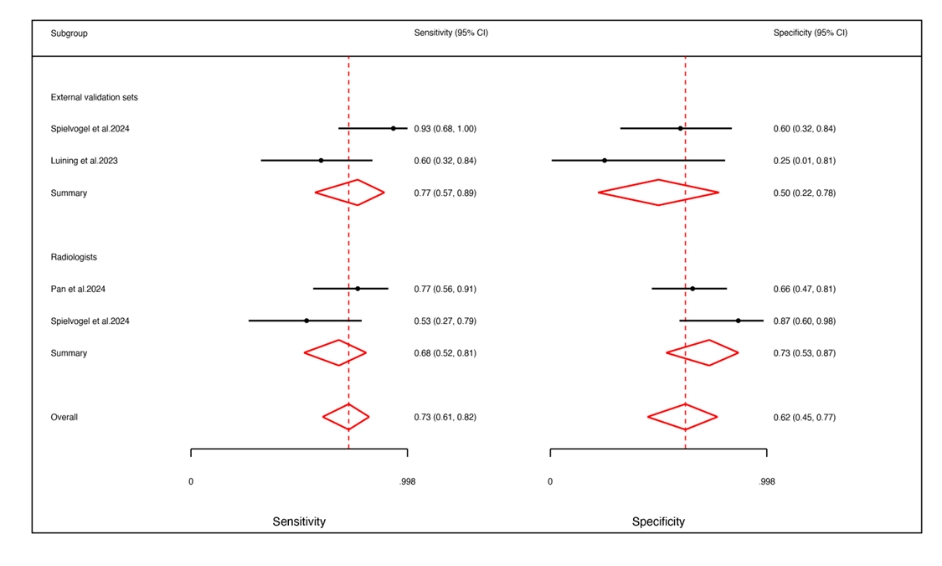
Figure S9: Subgroup forest plot of the diagnostic performance of PSMA-PET-based AI external validation sets compared to radiologists for predicting preoperative extraprostatic extension in prostate cancer. The sensitivity and specificity of each study were represented by squares, with horizontal bars indicating the 95% confidence intervals.

Figure S10: Deek's funnel plot of diagnostic performance of mpMRI based-artificial intelligence external validation sets for predicting preoperative extraprostatic extension in prostate cancer. *P<*0.05 was considered significant.


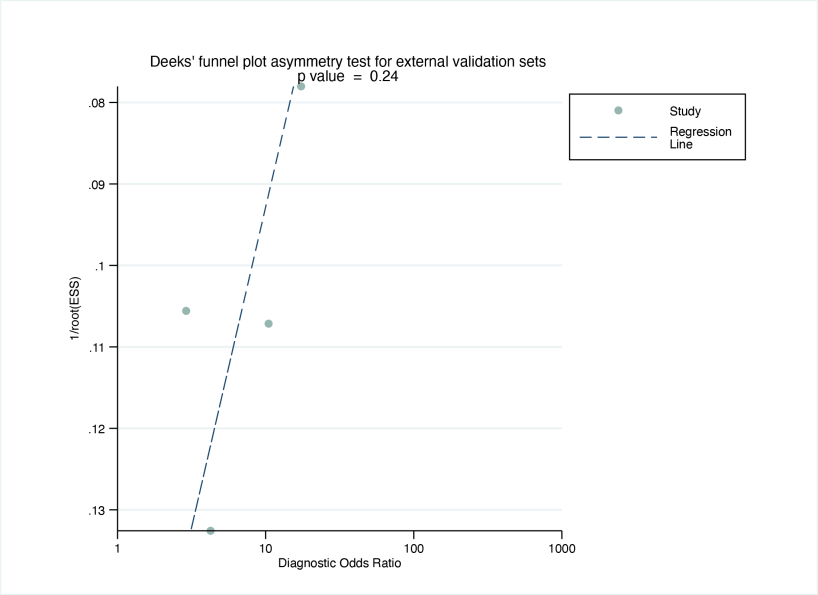


Figure S11: Deek's funnel plot of diagnostic performance of PSMA-PET based-artificial intelligence internal validation sets for predicting preoperative extraprostatic extension in prostate cancer. *P<*0.05 was considered significant.


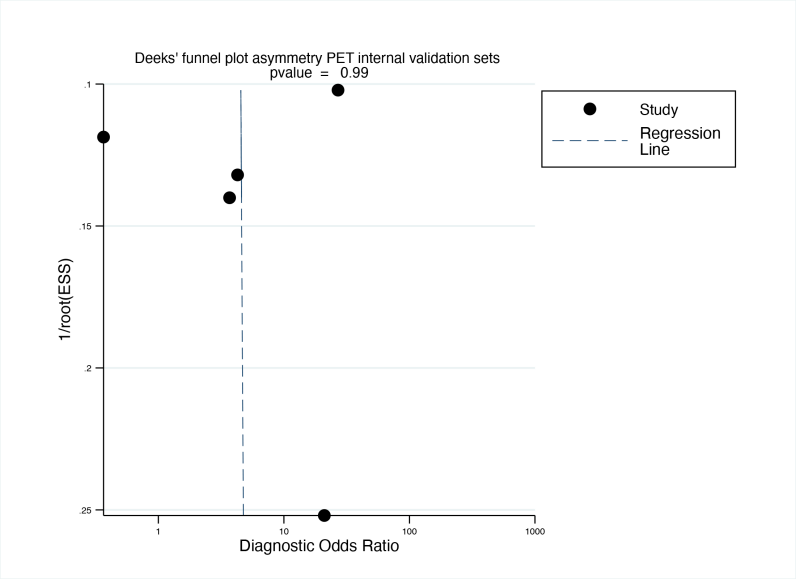

Supplement: Multimedia Appendix 1 [file jmir-v27-e80981-s001.docx]
